# Dynamic roles of ILC3 in endometrial repair and regeneration

**DOI:** 10.1093/discim/kyaf004

**Published:** 2025-03-26

**Authors:** Antonia O Cuff, Ee Von Woon, Thomas Bainton, Brendan Browne, Phoebe M Kirkwood, Frances Collins, Douglas A Gibson, Philippa T K Saunders, Andrew W Horne, Mark R Johnson, David A MacIntyre, Victoria Male

**Affiliations:** Department of Metabolism, Digestion and Reproduction, Imperial College London, London, UK; Wellcome Sanger Institute, Wellcome Genome Campus, Hinxton, UK; Department of Metabolism, Digestion and Reproduction, Imperial College London, London, UK; Department of Metabolism, Digestion and Reproduction, Imperial College London, London, UK; Department of Metabolism, Digestion and Reproduction, Imperial College London, London, UK; Centre for Reproductive Health, Institute of Regeneration and Repair, The University of Edinburgh, Edinburgh, UK; Centre for Reproductive Health, Institute of Regeneration and Repair, The University of Edinburgh, Edinburgh, UK; Centre for Reproductive Health, Institute of Regeneration and Repair, The University of Edinburgh, Edinburgh, UK; Centre for Reproductive Health, Institute of Regeneration and Repair, The University of Edinburgh, Edinburgh, UK; Centre for Reproductive Health, Institute of Regeneration and Repair, The University of Edinburgh, Edinburgh, UK; Department of Metabolism, Digestion and Reproduction, Imperial College London, London, UK; Department of Metabolism, Digestion and Reproduction, Imperial College London, London, UK; March of Dimes Prematurity Research Centre, Imperial College London, London, UK; Robinson Research Institute, University of Adelaide, North Adelaide, Australia; Department of Metabolism, Digestion and Reproduction, Imperial College London, London, UK

**Keywords:** innate lymphoid cell, mucosal immunology, reproductive immunology, endometriosis, endometrium

## Abstract

**Background:**

Innate lymphoid cells (ILCs) are prominent in the human uterine mucosa and play physiological roles in pregnancy. ILC3 are the second-most common ILC subset in the uterine mucosa, but their role remains unclear.

**Methods:**

Here we define two subsets of lineage-negative CD56+ CD117+ CRTH2-uterine ILC3, distinguished by their expression of CD127.

**Results:**

The CD127− subset is most numerous and active during menstruation and immediately after parturition, suggesting a role in the repair of the uterine mucosa (called endometrium outside of pregnancy); the CD127+ subset is most numerous and active immediately after menstruation, as the endometrium regenerates. In healthy endometrium, ILC3 are spatially associated with glandular epithelial and endothelial cells, which both express receptors for the ILC3-derived cytokines, IL-22 and IL-8. In the eutopic endometrium of people with endometriosis, ILC3 are located further from glandular epithelial and endothelial cells suggesting that these cells may be less exposed to ILC3 products, potentially with negative consequences for endometrial regeneration.

**Conclusion:**

Our findings highlight the dynamic nature of ILC3 in the uterine mucosa and suggest their primary role is in repair and regeneration. An improved understanding of uterine ILC3 will inform future research on endometrial health and disease.

## Graphical Abstract

**Graphical Abstract:**
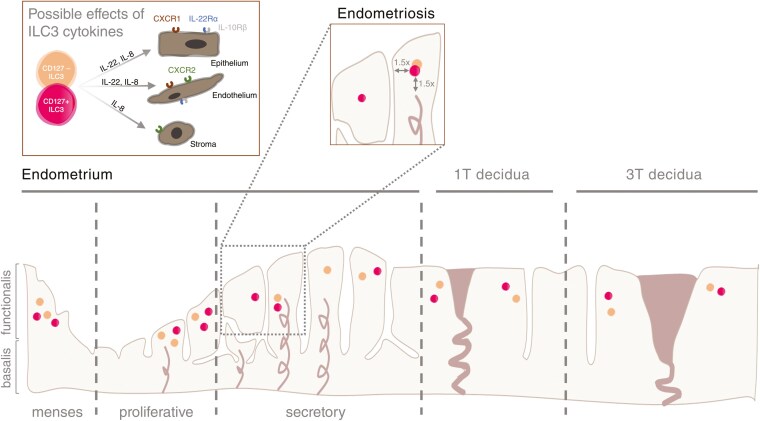

## Introduction

Innate lymphoid cells (ILCs) have essential roles at mucosal sites, contributing to barrier immunity and mucosal homeostasis. The ILC lineage broadly consists of three cell types: ILC1 (which is sometimes considered to include NK cells), ILC2, and ILC3 [1]. Uterine NK (uNK) cells are prominent in the human uterine mucosa, particularly in the first trimester of pregnancy, and are thought to have a role in placental implantation [2]. Previous studies have not detected ILC2 in human uterine mucosa [3, 4] while ILC3 have been reported in the non-pregnant uterine mucosa (endometrium), and first and third trimester pregnant uterine mucosa (decidua) [3–8]. However, the functions of ILC3 in human reproduction are yet to be elucidated.

A major challenge in studying ILC3 has been the diverse markers used to identify them [9, 10]. Across tissues, the most widely used definition is by no expression of markers of other immune cell lineages, positive expression of CD127 (IL-7Rα, a pan-ILC marker), no expression of CRTH2 (an ILC2 marker), and positive expression of CD117 (a marker of early lymphocyte development). ILC3 are sometimes subdivided into those that do (natural cytotoxicity receptor; NCR+) or do not (NCR−) express NK cell markers, including CD56. Uterine ILC3 have largely been identified as CD56+ [3, 5–7] and because of this, some studies of CD56+ cells that purportedly focus on uNK cells also include CD56+ uterine ILC3 [11]. Lymphoid tissue inducer (LTi) cells are related to ILC3 but are universally NCR− and CD56− LTi-like cells have also been reported in the decidua [5].

Human uterine ILC3 produce the prototypical cytokine IL-22 [5, 6], which supports epithelial cell homeostasis, particularly in the presence of bacterial infection [12]. Since trophoblast cells are epithelial, this suggests that ILC3 might also have a role in protecting against intrauterine infection during pregnancy. In support of this, IL-22 protects against LPS-induced pregnancy failure in mice [13], although, during infection with the intracellular bacteria Ureaplasma, IL-22 was found to increase the rate of preterm birth [14]. Although not a prototypical ILC3 cytokine, uterine ILC3 also produce IL-8 [5, 15], which has been proposed to play a role in ILC3-neutrophil crosstalk [15] and extravillous trophoblast (EVT) chemotaxis [16] associated with placental implantation.

Here, we describe a method to identify ILC3 subsets in the human uterine mucosa by flow cytometry and high dimensionality reduction analysis and use this to define the number and function of ILC3 subsets across the menstrual cycle, pregnancy, and the post-partum period. Uterine ILC3 numbers are highly dynamic, peaking at times of endometrial repair and regeneration. We examine their production of IL-8 and IL-22, since these may be relevant in physiological and pathological pregnancy and report that the production of these cytokines also peaks at times of repair and regeneration. We also find differences in ILC3 localization in the eutopic endometrium of people with endometriosis, compared to those without endometriosis. Collectively, our findings suggest a primary role for ILC3 in endometrial repair and regeneration and potentially implicate these cells in the pathogenesis of endometriosis.

## Materials and methods

### Human tissue

The collection of fresh human tissue was approved by the London Chelsea Research Ethics Committee (study numbers 10/H0801/45 for endometrium and 11/LO/0971 for decidua). Samples of the uterine mucosa were collected at different stages of the menstrual cycle and pregnancy (menses endometrium; proliferative endometrium; secretory endometrium; first trimester decidua; third trimester decidua; and post-partum endometrium) together with matched peripheral blood samples. Functionalis endometrium was collected as pipelle biopsies from healthy donors with regular cycles attending the sexual health clinic for a contraceptive coil fitting. The menstrual cycle phase was confirmed by the donor’s self-reported cycle day in combination with serum progesterone levels against a reference dataset [45]. Decidual samples from weeks 6–15 were obtained during pregnancy terminations upon personal request by the donor. Non-labouring decidual samples in third trimester pregnancies were collected at term (weeks 37–40) during elective caesareans.

Formalin-fixed and embedded pipelle biopsies taken at the time of surgery were accessed through the EXPPECT Biobank (Lothian Research Ethics Committee 11/AL/0376). In this cohort, controls were fertile patients being investigated for pelvic pain, but for whom no pathology was found. Donor demographics are shown in Supplementary Tables S1–S4.

Fresh pipelle endometrial biopsies were mechanically disaggregated through a 100 μm cell strainer, centrifuged (700×g, 10 min, RT) and resuspended in 10% FBS-supplemented PBS. Filtrates underwent repeat filtration through a 70 μm strainer and leukocytes were separated by density gradient centrifugation over Histopaque (700×g, 20 min, RT without brake).

First trimester decidual samples were processed as described [20]. Briefly, tissues were rinsed in PBS to remove excess blood and most of the placenta was removed by excision. Using scalpel blades, decidual tissues were minced and then homogenized in 10% FBS-supplemented PBS on a GentleMACS processor (Miltenyi). The homogenate underwent further mechanical disaggregation through a 75 μm sieve with regular washes in PBS. Filtrates were centrifuged (500×g, 10 min, RT), resuspended in 10% FBS-supplemented PBS and underwent density gradient centrifugation over Histopaque (700×g, 20 min, RT, without brake).

Decidua basalis and parietalis were dissected from the term placentae as described [46]. To isolate leukocytes and placental cells, tissues were mechanically disaggregated by GentleMACS homogenization in 1 ml Accutase per gram tissue. Homogenized tissue was then digested (100 rpm, 1 h, 37°C), digests were filtered through a 70 μm strainer and leukocytes were separated by density gradient centrifugation over Histopaque (700×g, 20 min, RT, without brake). Non-immune cells other than placental cells were isolated as described [47]. Dissected tissues were cleansed of blood in PBS and homogenized in 0.25% trypsin-enriched PBS containing 0.05% DNAse I. Tissues were enzymatically disaggregated (100 rpm, 15 min, 37°C) before trypsin activity was quenched in serum-rich media (10% newborn calf serum-supplemented DMEM/F12 medium, plus 1% penicillin-streptomycin and 0.4% DNase I). Digests were filtered through a gauze mesh, centrifuged (500xg, 20 min, RT), resuspended in serum-rich media, refiltered through a 100 μm strainer and overlayed on Histopaque for cell isolation by density gradient centrifugation (700×g, 20 min, RT, without brake).

### Flow cytometry

Viable cells were identified using fixable viability stain eFluor 450 (eBioscience) in PBS (15 min, 4°C). Non-specific antibody staining was inhibited using blocking buffer (male 10% human AB serum, 1% bovine serum albumin, 2 mM EDTA, 25 µg BD Fc Block; 15 min, 4°C) and washed before completing surface stain in staining buffer (PBS supplemented with 1% FBS, 2 mM EDTA). Brilliant stain buffer (BD Biosciences) was added to the surface stains to counteract anticipated intra-brilliant violet fluorescence interferences. Intracellular antigens were stained using the Human Foxp3 Fix/Perm buffer (BD Biosciences). Surface-stained leukocytes were fixed (10 min, RT), washed then permeabilized (30 min, RT) before intracellular staining antibodies (Supplementary Table S5) were added (30 min, RT). Washes at the end of each step were completed in the staining buffer. Data were acquired on a BD LSR Fortessa X-20 cytometer. Details of antibodies are given in Supplementary Table S5.

Immune cell frequencies and mean fluorescence intensities were extracted using FlowJo (Treestar). Normalized immune cell counts were generated by multiplying the frequency of each cell type (as a proportion of live CD45+ cells) by the absolute number of immune cells isolated per gram of tissue. High dimensionality reduction analyses were also completed in FlowJo, following data preparation in line with the current recommendations for optimal cytometry experiments, eliminating anomalies such as fluidic errors, doublets, and dead cells [48, 49].

### Functional assays

For staining of effector molecules, cells were cultured for 4 h in RPMI-supplemented media (RPMI 1640—Glutamax, 10% foetal bovine serum, penicillin-streptomycin, 1× non-essential amino acids, 1 mM sodium pyruvate, 25 mM HEPES, and 50 μM B-mercaptoethanol), and in the presence of Brefeldin (10 μg/ml) and Monensin (2 μM). Cells were cultured either without stimulation or with non-specific stimulation with phorbol myristate acetate (PMA, 50 ng/ml, Sigma) and ionomycin (1 μg/ml, Sigma).

### Immunofluorescence microscopy

Endometrial tissue sections (5 μm thick) were dewaxed in xylene and rehydrated in serial ethanol dilutions. Antigens were unmasked by sequential antigen retrieval as in Supplementary Table S6a. Tissues were permeabilized in 0.25% Triton in PBS (30 min, RT). Non-specific antigen detection was achieved by incubation in 3% hydrogen peroxide (30 min, RT) and serum-rich block (5% normal donkey serum, 2% bovine serum albumin in PBS, 30 min, RT). Primary antibodies (Supplementary Table S6a) attached overnight at 4°C and after washes in 0.05% Tween-20-supplemented PBS were detected using HRP-conjugated secondary antibodies (Supplementary Table S6b) and HRP-activated dyes (Supplementary Table S6c). Tissues were mounted in DAPI and imaged. Fields of view for imaging were randomly selected, from among fields of view containing at least one CD56+ CD117+ cell. Images were captured on a Zeiss Axioscan Z1 fluorescence microscope, processed in ZEN Blue and processed images were analysed in FIJI.

### Statistical analysis

Data were tested for normality using Shapiro–Wilk’s test, to inform downstream choice of statistical testing. GraphPad Prism was used to plot data and perform statistical tests. Details of the specific test used in each case are given in the corresponding figure legends.

## Results

### Identification of ILC3 subsets in human uterine mucosa

**Figure 1.**
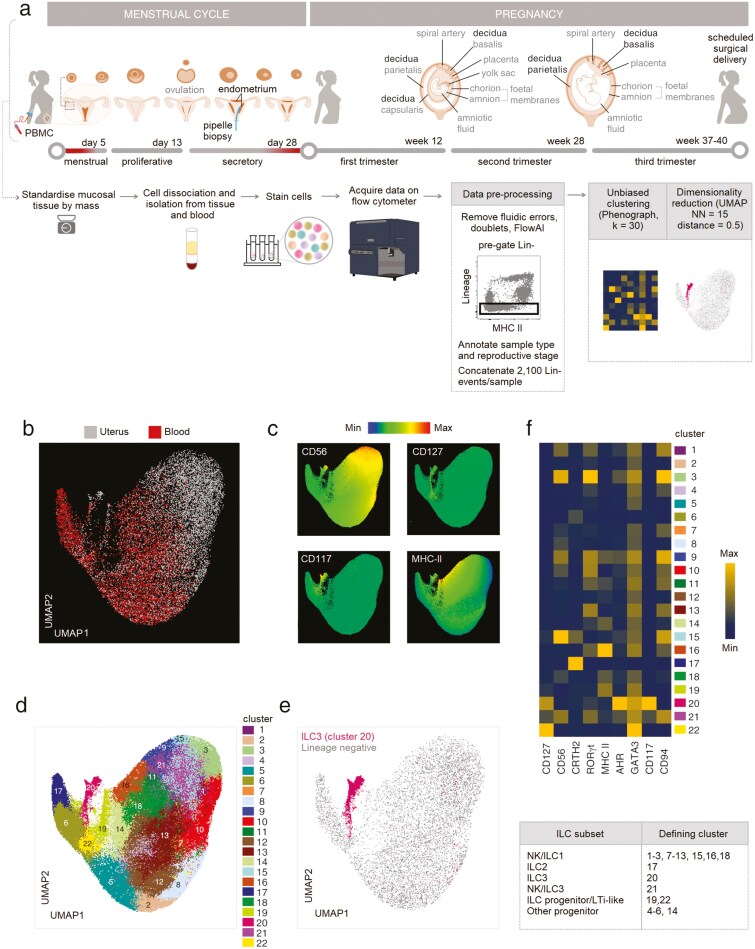
Identification of ILC3 subsets in human uterine mucosa. (A) Schematic illustration of the stages of human reproduction, anatomical locations of specific types of uterine mucosa analysed (bold font) and workflow on uterine mucosal and matched peripheral blood samples. (B) Single, live, CD45+ lineage– (CD3, CD4, CD14, CD19, and CD34) cells are distributed across the UMAP and coloured by origin (uterine mucosa, grey, 132,000 cells; blood, red, *n* = 108,000 cells). (C) Relative intensity of expression of the indicated markers is shown. (D) Unbiased clustering of CD45+ lin– cells was completed using phenograph. (E) Cluster 20, with a phenotype consistent of ILC3, is highlighted in deep pink. (F) The heatmap shows the geometric mean fluorescence intensities of the indicated markers in each cluster. Menses (M, *n* = 7), proliferative (P, *n* = 7), secretory (S, *n* = 7), first trimester (1T, *n* = 12), third trimester (3T blood, *n* = 8), decidua basalis (3B, *n* = 8) or parietalis (3P, *n* = 8), and post-partum (PP, *n* = 5).

Because there is currently no consensus definition of ILC3 across uterine mucosal tissues, we examined cells of the uterine mucosa at different stages of the menstrual cycle and pregnancy (menses endometrium, 7; proliferative endometrium, 7; secretory endometrium, 7; first trimester decidua, 12; third trimester decidua, 8; post-partum endometrium, 5) together with matched peripheral blood cells, by flow cytometry (Fig. 1a). Endometrial samples were collected from healthy donors attending for contraceptive coil fitting, first trimester samples from terminations of pregnancy at the person’s request, and third trimester samples from non-emergent caesarean delivery. We examined CD45+ lineage-negative (CD3, CD4, CD14, CD19, and CD34) cells (Supplementary Fig. S1a) using an unsupervised approach. UMAP analysis revealed two major regions of cells: those originating from the uterine mucosa (grey) and those derived from the blood (red) (Fig. 1b), consistent with previous reports [7]. There was some overlap between uterine and peripheral blood ILC subsets, suggesting that some subsets share similar phenotypes in both compartments. Cells expressing high levels of CD56 were overrepresented in the uterine mucosa (Fig. 1c), likely to include uterine NK cells, other CD56+ ILC1 [7] and CD56+ ILC3 [3, 5, 6].

To define ILC3, cells were clustered using Phenograph and those expressing ILC3 markers, including AHR, RORγt, CD117, CD127, and MHC II [9, 17], were identified (Fig. 1c). Cluster 20 expressed the ILC lineage-defining transcription factors AHR and RORγt, as well as high levels of CD117 and bimodal levels of CD127 (Fig. 1c–f; Supplementary Fig. S1b). MHC II, associated with antigen presentation and immune cell activation, was expressed in cluster 20, as well as adjoining regions. Identities were assigned to other clusters based on the expression of canonical markers, shown in Fig. 1d and f.

**Figure 2.**
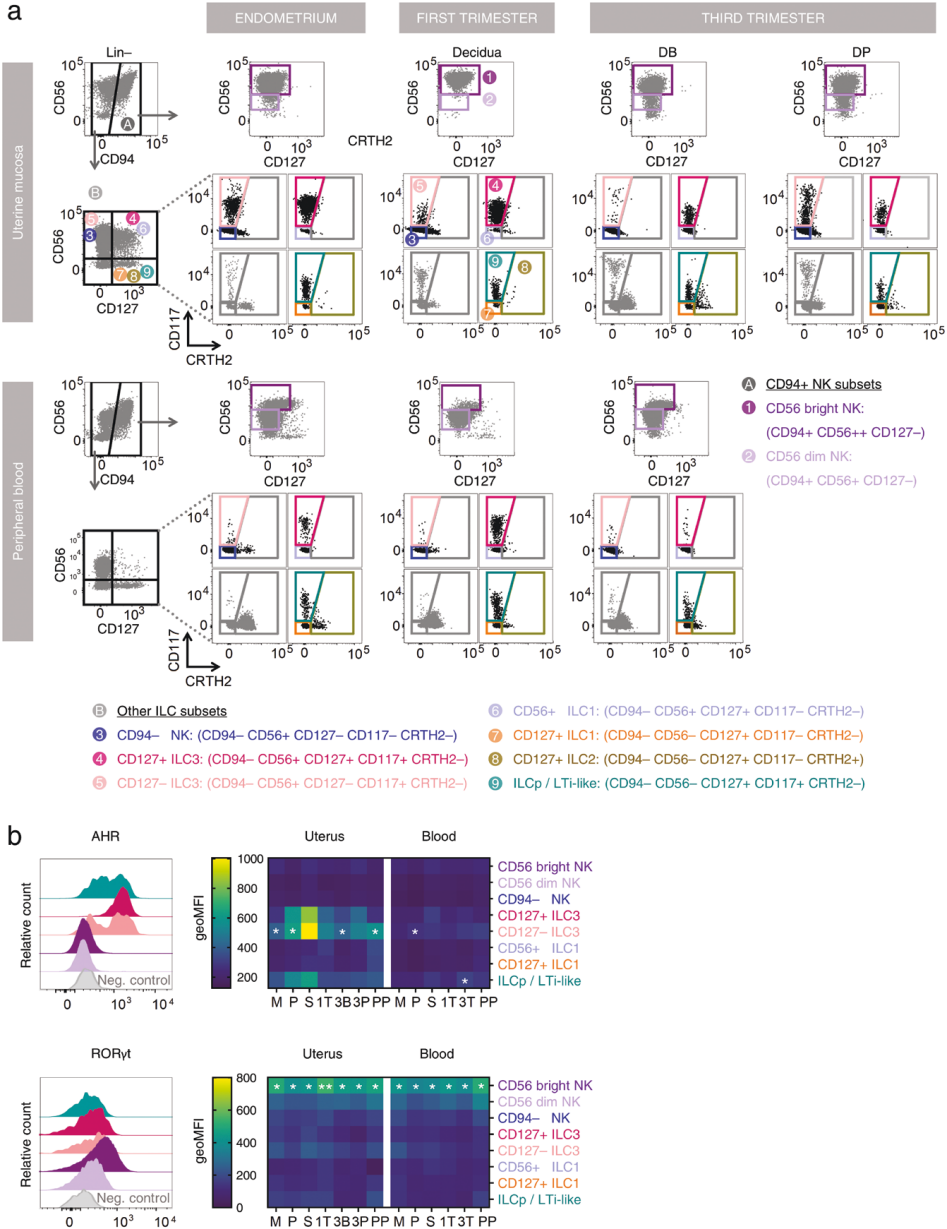
Uterine ILC3 are present throughout the menstrual cycle, pregnancy and post-partum. (A) Flow cytometric gating strategy to identify ILC subsets among CD45+ lin– immune cells isolated from uterine mucosal tissue and matched peripheral blood at different stages of the menstrual cycle, pregnancy and post-partum. (B) Relative fluorescence intensities of staining for the ILC3 lineage-defining transcription factors, AHR and RORγt across the menstrual cycle and pregnancy. Menses (M, *n* = 7), proliferative (P, *n* = 7), secretory (S, *n* = 7), first trimester (1T, *n* = 12), third trimester (3T blood, *n* = 8), decidua basalis (3B, *n* = 8) or parietalis (3P, *n* = 8), and post-partum (PP, *n* = 5). Statistical significance was determined by normalization using *Z* score transformation (one-tailed, * *P* < 0.05, ** *P* < 0.01).

We next used these features to design a conventional flow cytometric gating strategy to identify ILC3 in reproductive tissues. Lineage-negative CD94− CD127+ CD117+ CD56+, as previously described in decidua [5, 6] and secondary lymphoid tissues [18], were consistently identified in uterine mucosa spanning the menstrual cycle and pregnancy (Fig. 2a). The lineage-defining transcription factors RORγt and AHR were expressed by these cells (Fig. 2b). The bimodal distribution of CD127 in cluster 20 also prompted us to examine lineage-negative CD94− CD127− CD117+ and CD56+ cells (Fig. 2a). The expression of RORγt and AHR by these cells (Fig. 2b) suggests that these are also ILC3: therefore, these CD127− ILC3 were also included in the downstream analyses of ILC3 dynamics in the human uterine mucosa.

Lineage-negative CD94− CD127+ CD117+ CD56− cells are classified as ILC progenitors (ILCp) in the blood [19] but have also been called LTi-like cells in the decidua [5]. We identified cells with this phenotype in blood, decidua, and endometrium (Fig. 2a). As we did not assess either the ILC progenitor or LTi potential of these cells, they are hereafter referred to as ‘ILCp/LTi-like cells’. These cells expressed AHR but at lower levels than the ILC3 subsets (Fig. 2b).

Of note, and in contrast to previous reports [3, 4], we were able to identify a small subset of lineage-negative CD127+ CRTH2+ cells in endometrium and decidua, which is likely to represent ILC2.

### Uterine ILC3 are most numerous and active at times of endometrial repair and regeneration

**Figure 3.**
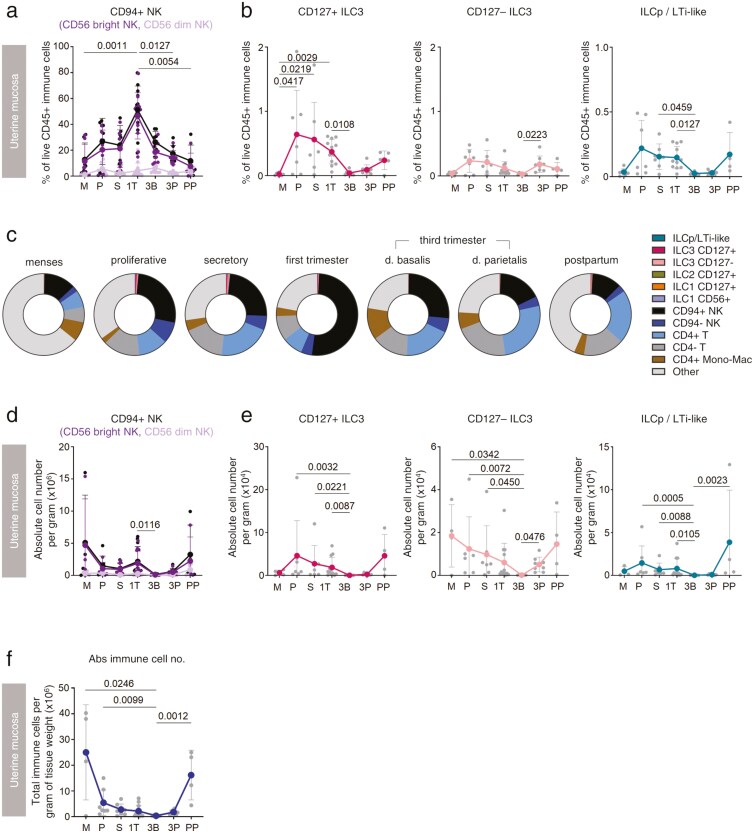
Uterine ILC3 are most numerous at times of tissue repair and regeneration. (A) Frequency of CD94+ NK cells in the uterus across the menstrual cycle and pregnancy (CD56 bright and CD56 dim subpopulations of total CD94+ NK cells are also shown). (B) Frequency of ILC3 subsets in uterine mucosa across the menstrual cycle and pregnancy. (C) Representative frequencies of all live CD45+ immune cell populations in the uterus at different stages of the human reproductive life cycle. (D) Absolute number of CD94+ NK cells per gram of tissue. (E) Absolute number of ILC3 subsets per gram of tissue. (F) Absolute number of all live CD45+ immune cells per gram of tissue. A Kruskal–Wallis statistical test for significance was performed with a Dunn’s correction for multiple hypotheses testing (* *P* < 0.05, ** *P* < 0.01). Menses (M, *n* = 7), proliferative (P, *n* = 7), secretory (S, *n* = 7), first trimester (1T, *n* = 12), third trimester (3T blood, *n* = 8), decidua basalis (3B, *n* = 8) or parietalis (3P, *n* = 8), and post-partum (PP, *n* = 5).

We next traced the distribution of ILC3 in uterine mucosal samples from healthy participants across reproductive stages. The frequency of uNK cells was used to validate immune cell isolation methods. Consistent with previous reports, their number and frequency peaked during the first trimester of pregnancy [20] (Fig. 3a and d).

In contrast, CD127+ ILC3, CD127− ILC3, and ILCp/LTi-like cells were most frequent in the proliferative and secretory phases of the menstrual cycle and in the post-partum period, together accounting for approximately 1% of all immune cells during their peak at the proliferative phase of a menstrual cycle (Fig. 3b and c). Cell frequencies can decrease due either to a decrease in their number or an increase in the number of other CD45+ cells, so we also determined the absolute number of each subset per gram of tissue (Fig. 3d and e). CD127+ ILC3 and ILCp/LTi-like cells were most numerous in regenerating proliferative and post-partum endometrium while CD127− ILC3 were most numerous during menstruation and in the post-partum period, times of endometrial repair. For all subsets, the fewest cells were observed during the third trimester of pregnancy. Fluctuations in the total number of immune cells are shown for context (Fig. 3f). Since there is variation in uterine mucosal immune cell number throughout the secretory phase, we also present the data divided by early, mid, and late secretory sub-stages (Supplementary Fig. S2a–d). In this analysis, there are few samples in each stage, making statistical significance hard to define, but we note a downward trend in the numbers and frequencies of both CD127+ and CD127− ILC3 subsets as the secretory phase progresses.

In the peripheral blood, few significant changes were observed, although there was a trend towards fewer CD127+ ILC3 and ILCp/LTi-like cells in the third trimester and post-partum (Supplementary Fig. S2e–h).

**Figure 4.**
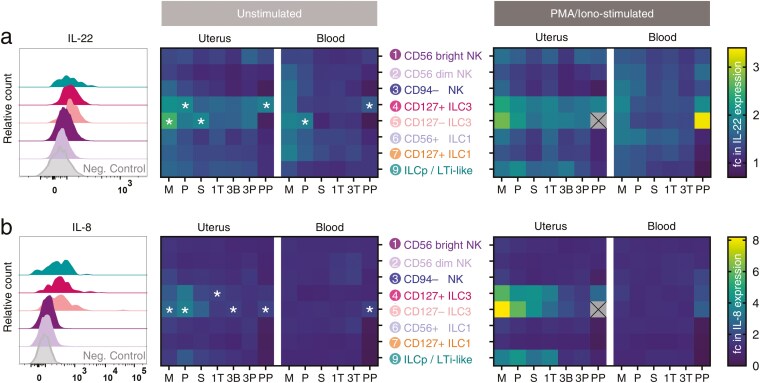
Uterine ILC produce IL-22 and IL-8 preferentially at times of tissue repair and regeneration. (A and B) Isolated leukocytes were cultured directly *ex vivo* in the presence of Brefeldin A (10 µg/ml) and Monensin (2 µM) for 4 h. Phorbol myristate acetate (PMA, 50 ng/ml) and ionomycin (1 µg/ml) allowed the examination of effector molecule production by ILCs for the stimulation condition. Values are normalized to an internal negative control (CD4− CD56− MHC II− T cells for IL-22, CD4+ CD56− MHC II− T cells for IL-8; for gating strategy see Supplementary Fig. S3). Statistical significance was determined by Z score transformation (one-tailed, * *P* < 0.05). Histograms and heatmaps for (A) IL-22 and (B) IL-8 expression by ILC subsets across the menstrual cycle and pregnancy are shown. Stages examined included the menses (M, *n* = 5), proliferative (P, *n* = 6 uterus, *n* = 5 blood), secretory (S, *n* = 7), first trimester (1T, *n* = 11 uterus, *n* = 10 blood), third trimester (3T blood, *n* = 5), decidua basalis (3B, *n* = 5) or parietalis (3P, *n* = 5), and post-partum (PP, *n* = 3) stages.

We next examined the capacity of uterine ILC3 and other immune cells identified as a byproduct of the flow panel to produce IL-22 and IL-8. ILC3 subsets in the uterine mucosa produced more IL-22 and IL-8 than other ILC subsets, with CD127+ and CD127− ILC3, rather than ILCp/LTi-like cells, the major producers of these cytokines. IL-22 was produced by uterine ILC3 subsets across all reproductive stages, but unstimulated CD127+ and CD127− ILC3 displayed significantly higher production of IL-22 during early menstrual cycle and post-partum phases (Fig. 4). A small subset of CD3+ CD4+ CD56+ T cells, likely to represent iNKT, also produced significant IL-22, although the very low numbers of these cells suggest they are unlikely to contribute to IL-22 production more than ILC3 (Supplementary Fig. S3).

IL-8 production by CD127− ILC3 was also highest in the early menstrual cycle and immediately post-partum, although there was also some IL-8 production in the third trimester decidua basalis. Myeloid-lineage cells produced high levels of IL-8 (Supplementary Fig. S3) and given their greater abundance than ILC3 are therefore likely to be the major IL-8-producing immune cell subset in the uterine mucosa; non-immune cells, notably epithelial cells, have also previously been reported as a major source of IL-8 in the endometrium [21].

MHC II, which presents antigen and is a marker of immunological activation, was more highly expressed by ILC3 towards the end of pregnancy (Supplementary Fig. S4).

### IL-22 and IL-8 receptors are expressed by epithelial and endothelial cells

**Figure 5.**
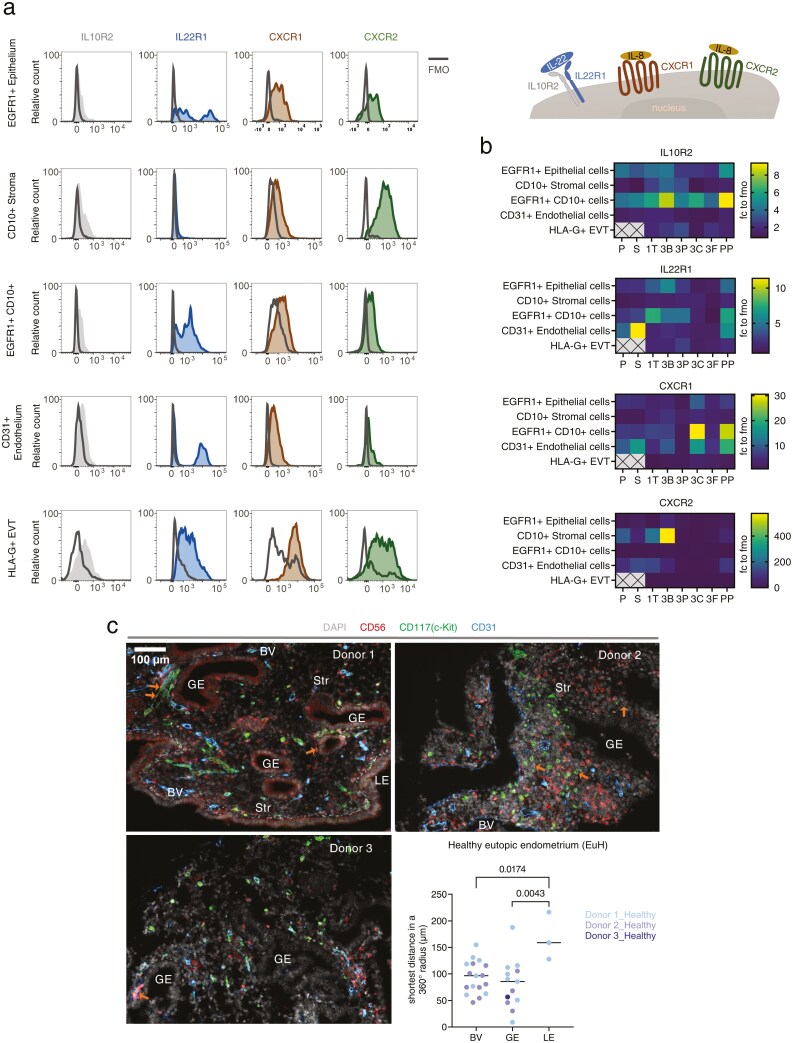
ILC3 are located near endothelial and glandular epithelial cells, which express IL-22 and IL-8 receptors. (A) Representative histograms of IL10R2, IL22R1, CXCR1, and CXCR2 relative to the fluorescence minus one (FMO) control in different cellular components of the uterine mucosa. (B) Summarized heatmap of receptor expression, normalized to the FMO control. Proliferative (P, *n* = 2), secretory (S, *n* = 4), first trimester (1T, *n* = 10), third trimester decidua basalis (3B, *n* = 7), decidua parietalis (3P, *n* = 7), chorionic membrane (3C, *n* = 2), foetal placenta (3F, *n* = 2), and post-partum (PP, *n* = 2). (C) Spatial location of ILC3 in the functional layer of endometrium from three healthy donors are shown through confocal microscopy. Markers included CD56 (red), CD117 (green), CD31 (blue), and DAPI staining for the nucleus (grey). Orange arrows pinpoint ILC3, defined as CD56+ CD117+. The distance between each ILC3 and their surrounding endometrial structural component were determined using Fiji and statistically significant differences are indicated (one-way nested ANOVA, two-tailed, median shown).

To determine which cell types could be responding to ILC3-derived IL-22 and IL-8, we next examined the expression of the receptors for these cytokines. The expression of the IL-22 receptor (a dimer of IL10R2 and IL22R1) and IL-8 receptors CXCR1 and CXCR2 was examined on CD45− cell subsets identified as shown in Supplementary Fig. S5. The expression of each receptor was normalized to the fluorescence minus one (FMO) control (Fig. 5a).

Of the two IL-22 receptor subunits, IL10R2 was more widely expressed than IL22R1 across reproductive stages (Fig. 5b). EGFR1+ CD10−epithelial cells and EGFR1+ CD10+ cells highly express both IL10R2 and IL22R1. CD31+ vascular endothelial cells uniformly express a low level of IL10R2 while a subset also express IL22R1. During pregnancy, EVT expresses both subunits of the IL-22 receptor, albeit at a low level compared to the FMO control.

Some expression of the IL-8 receptors CXCR1 and CXCR2 was observed across all cell types examined (Fig. 5a) but their expression was more variable across reproductive stages than that of the IL-22 receptor subunits (Fig. 5b). The highest levels of CXCR1 expression were observed in CD31+ vascular endothelial and EGFR1+ CD10+ cells isolated from third trimester chorionic membrane, and from post-partum endometrium. In contrast, CXCR2 was more highly expressed in CD10+ stromal cells, particularly those isolated from third trimester decidua basalis.

### Uterine ILC3 are located near blood vessels and glandular epithelium

To investigate the propensity for interaction between uterine ILC3 and these non-immune cell types, we next sought to establish their proximity to each other. We focused on the endometrium since ILC3 were most numerous and active at this reproductive stage, examining biobanked endometrial samples from donors being investigated for pelvic pain who were not found to have a gynaecological pathology (hereafter referred to as ‘healthy donors’). Multiplex immunofluorescence microscopy showed that ILC3 markers such as RORγt, AHR, and CD127, which have been previously used on frozen tissue [22–24], were masked by fixation. Therefore, we identified ILC3 as CD56+ CD117+, indicated by orange arrows (Fig. 5c; Supplementary Fig. S6). The majority of CD56+ CD117− cells represent uNK cells, although this group may also include some CD56+ innate T cells. Vascular endothelial cells (labelled BV) were identified by staining for CD31. Other cells were identified by morphology: luminal epithelial cells (a wall of columnar epithelia; LE), glandular epithelial cells (a mixture of cells with epithelial cell morphology largely gathered around a central cavity; GE), and stromal cells (the cell network which makes up the bulk of the endometrial structure and lies between the glands and blood vessels; Str).

CD56+ CD117+ ILC3 were found in the endometrium of all three healthy donors examined, although they were less frequent than CD56+ CD117− uNK cells, consistent with our observations by flow cytometry. ILC3 were located within the stroma, but close to blood vessels and glands. Quantification of the shortest distance between ILC3 and different endometrial components revealed that ILC3 were located at approximately equivalent distances from blood vessels and glands, but significantly further away from the luminal epithelium (Fig. 5c). As a comparison, we examined the location of CD56+ CD117− uNK: this increased propensity for association with blood vessels and glands, compared to the luminal epithelium, was not observed for NK cells (Supplementary Fig. S7c).

### ILC3 are located further from blood vessels and glandular epithelium in endometriosis

Our observation that ILC3 are most abundant and active at times of endometrial regeneration suggested a role in this process. Furthermore, our findings that ILC3 are most closely associated with blood vessels and glands and that vascular endothelial and epithelial cells express receptors for their products, are consistent with interactions between ILC3 and these cells. Therefore, we sought to investigate whether the dynamics and localization of ILC3 differed between healthy donors and those suffering from menstrual cycle disorders. We focused on endometriosis because of the well-established role of immune dysregulation in the pathology of the disease [25, 26], including a recent report that ILCs are reduced in the endometrium during endometriosis [27].

**Figure 6.**
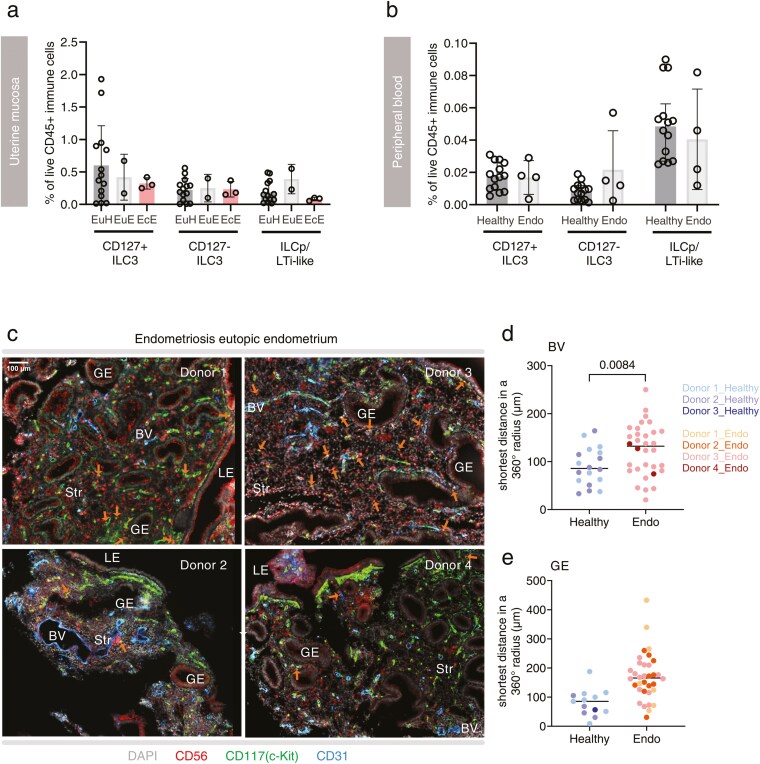
Endometrial ILC3 are located further from endothelial and glandular cells in endometriosis. (A) Frequency of ILC3 subsets among total live CD45+ immune leukocytes isolated from eutopic endometrium in healthy donors [EuH, proliferative (P, *n* = 7)], secretory (S, *n* = 7)], eutopic endometrium in donors with endometriosis [EuE, proliferative (P, *n* = 1), secretory (S, *n* = 1)], and ectopic endometrium in donors with endometriosis [EcE, proliferative (P, *n* = 1), secretory (S, *n* = 1), and undefined (*n* = 1)]. (B) Frequency of ILC3 subsets in the blood of healthy donors and donors with endometriosis healthy donors, proliferative (P, *n* = 7), secretory (S, *n* = 7); endometriosis donors, proliferative (P, *n* = 1), secretory (S, *n* = 2), and undefined (*n* = 1). (C) Spatial location of ILC3 in the functional layer of eutopic endometrium from four endometriosis patients are shown through confocal microscopy. Markers included CD56 (red), CD117 (green), CD31 (blue), and DAPI staining for the nucleus (grey). Orange arrows pinpoint ILC3, defined as CD56+ CD117+. (D and E) The distance between each ILC3 and their nearest (D) blood vessel or (E) glandular epithelial cell was determined using Fiji. Statistically significant differences between healthy donors (data duplicated from Fig. 5c) and endometriosis patients are indicated (nested *t*-test, two-tailed).

CD127+ ILC3 in the eutopic endometrium of donors with endometriosis (EuE) were less frequent than in eutopic endometrium from healthy donors (EuH), although the difference was not significant, and there was no difference in the frequency of CD127− ILC3 (Fig. 6a). In donors with endometriosis, the frequency of both ILC3 subtypes in the ectopic endometrium was comparable to their frequency in eutopic endometrium. ILCp/LTi-like cells tended towards a higher frequency in the eutopic endometrium of donors with endometriosis than in eutopic endometrium of healthy donors, although this difference was not significant. Peripheral blood ILC3 subtypes did not differ in donors with endometriosis relative to equivalent cells in healthy donors (Fig. 6b). The frequencies of other immune cells that could be identified using our gating strategy are shown in Supplementary Fig. S7a and b.

Next, we used multiplex immunofluorescence microscopy to examine the spatial distribution of ILC3 in the eutopic endometrium of donors with endometriosis compared to that of healthy donors (Fig. 5c). All donors were in the secretory phase of the menstrual cycle. In line with our findings by flow cytometry, ILC3 were detected in the eutopic endometrium of endometriosis patients by microscopy (Fig. 6c). These were located further away from blood vessels and glands than in controls, although the difference was only significant for blood vessels (Fig. 6d and e). Of note, no significant changes in localization were observed for CD56+ CD117−uNK (Supplementary Fig. S7d–f).

## Discussion

Group 1 ILCs in the form of uNK cells are well represented in the human uterine mucosa, while ILC2 are rare. Here we provide new insight into the dynamics of ILC3 across the menstrual cycle and pregnancy. Previous attempts to define the dynamics and behaviour of ILC3 in the human uterus have been hampered by the lack of a consistent method to identify these cells, as well as the extent to which ILC3 share markers with more prominent uNK cell populations. Using high dimensionality reduction analysis, we identified two ILC3 subsets in the human uterine mucosa, and an ILCp/LTi-like subset. The identity of the subsets was confirmed by their expression of AHR, RORγt, and IL-22 and all three subsets were detectable throughout the menstrual cycle and pregnancy. In line with a previous report that CD56^bright^ NK cells express RORγt in secondary lymphoid tissue [28], we also observed RORγt expression in CD56^bright^ uNK cells.

Across tissues, ILC3 are most often identified as lineage-negative CD127+ CD117+ CRTH2− [10]: noting that we defined the two ILC3 subsets as CD56+, one of these subsets therefore closely aligns with the definition of NCR+ ILC3 used in other tissues. Of note, the other ILC3 subset we identified expressed little of the canonical ILC marker, CD127. CD127- ILC2 have recently been reported [29] and have been proposed to arise when ILC2 downregulate CD127 in response to IL-7 signalling; CD127− ILC3 could potentially arise in the uterus by similar mechanisms. Alternatively, NCR+ ILC3 could downregulate RORγt, CD117, and CD127 becoming ILC1-like ‘ex-ILC3’ cells [10, 30]. An intriguing possibility is that the CD127− uterine ILC3 we describe here follow a similar trajectory: this could potentially explain their bimodal expression of the ILC3 lineage-defining transcription factor AHR. In this case, our finding that AHR expression in CD127− uterine ILC3 varies over the menstrual cycle and pregnancy could indicate a shift from ILC3 to ‘ex-ILC3’ mediated by the changing microenvironment.

Previous analyses of group 1 uterine ILC frequencies found that peak uNK cell frequency and function coincided with the physiological processes with which they are most closely associated: implantation and placentation [20]. Equipped with the ability to discern ILC3 subsets in the human uterine mucosa, we took a similar approach to determine the stage at which ILC3 are most active, and therefore when they are most likely to have a physiological role. One challenge of this approach is that the uterine mucosa is highly dynamic, with cyclic changes in other immune cell subsets impacting any analysis based only on cell frequencies: to mitigate this we also examined absolute changes in cell number per gram of tissue. Based on previous work in mice [13, 31] and humans [15], we expected that ILC3 might have a role in pregnancy. However, CD127− ILC3 were the most numerous and had the greatest potential to produce IL-22 and IL-8 at times of endometrial repair, during menstruation and in the immediate post-partum period. CD127+ ILC3 were most numerous and had the greatest potential to produce IL-22 in proliferative and post-partum endometrium, suggestive of a role in endometrial regeneration: our findings with respect to CD127+ ILC3 were similar to those in a recent report, which found ILC3 were more frequent in the proliferative than the secretory phase, although not significantly so [32].

Further to analysing ILC3 production of IL-22 and IL-8, we also looked at the propensity for target cells in the uterine mucosa to respond to these cytokines. In line with a previous study by immunohistochemistry [33], we found IL10R2 expression in the epithelial and stromal cell compartments, as well as in EGFR1+ CD10+ cells, which may represent stromal cells undergoing mesenchymal to epithelial transformation during endometrial repair [34]. IL22R1 was also expressed by these cells throughout the menstrual cycle, in contrast to the findings of an earlier immunohistochemistry-based study that detected IL22R1 only in secretory endometrium [33]. In our study, endothelial cells in the same stages prominently expressed IL22R1 but only expressed IL10R2 at low levels. We detected expression of the IL-8 receptors CXCR1 and CXCR2 among endothelial, stromal and stromal-epithelial intermediate cells, but little expression among epithelial cells. This is consistent with a previous study which detected CXCR1 and CXCR2 in endometrial stromal cells by immunohistochemistry, although this study also reported no expression of these receptors in endothelial cells, in contrast to our findings [35]. The differences between our findings and those previously reported may be accounted for by the fact that flow cytometry enables the analysis of a larger number of cells and is thus better able to detect small numbers of highly expressing endothelial cells.

Our analysis of the spatial distribution of ILC3 in the endometrium finds that they are more closely associated with blood vessels and glands than luminal epithelium. This supports the concept that ILC3 are most abundant deeper in the uterine mucosa, similar to their location at other mucosal sites [36]. Expression of IL-22 receptors by epithelial and endothelial cells, and the proximity between ILC3 and these cells is indicative of crosstalk between ILC3, endometrial glands, and blood vessels.

To investigate this further, we characterized the dynamics of ILC3 in the endometrium of donors with endometriosis, compared to healthy control donors. Two papers have recently reported on ILC3 in the endometrium of donors with endometriosis, with one finding ILC3 reduced in endometriosis patients compared to healthy donors [27], while the other found no significant difference, although the trend was towards a reduction [32]. Similarly, we also found CD127+ ILC3 less frequent in the eutopic endometrium of donors with endometriosis compared to healthy donors, although this difference was not statistically significant in our cohort. One important limitation of our examination of fresh tissue by flow cytometry was that we were unable to collect samples at a single stage of the menstrual cycle, meaning that variability across the cycle may mask differences between endometriosis patients compared to healthy donors.

Investigation of cycle-matched samples by microscopy showed that ILC3 in the endometrium from donors with endometriosis were located significantly further from the vasculature than those in controls and a similar trend was observed for ILC3 location compared to the glands. This raises the possibility that endothelial and glandular epithelial cells are less exposed to IL-22 in the eutopic endometrium of people with endometriosis. IL-22 is well-known to promote epithelial cell proliferation [37] and there is also some evidence it can act on endothelial cells to promote angiogenesis [38]. Both processes are important for the repair of the uterine mucosa, which occurs simultaneously with menstruation, and subsequent regeneration. Of note, people with endometriosis are approximately five times more likely to report heavy menstrual bleeding than controls [39]. Although it has been suggested that the comorbidity between endometriosis and heavy menstrual bleeding results from progesterone resistance and endometrial hyperplasia, this view has become increasingly contested [40] and there is some evidence that the major endometrial ILC subset, uNK cells, are dysregulated in heavy menstrual bleeding [41]. Our results are consistent with the concept that heavy menstrual bleeding in endometriosis could in part result from inadequate endometrial repair. Further studies determining whether other etiologies of heavy menstrual bleeding are also associated with differential distribution of endometrial ILC3 are warranted.

While our study reveals important new insight into ILC3 function in the uterine mucosa, it was limited to the examination of the upper layer of the endometrium (functionalis). The location of ILC3 in the deeper layer (basalis) remains to be determined. Furthermore, while the prominence of ILC3 in the non-pregnant endometrium suggests a physiological role outside of pregnancy, we do not rule out a role in pregnancy. Expression of IL-22 and IL-8 receptors by extravillous trophoblasts indicates that they stand ready to detect ILC3 products if they are produced, perhaps in pathological situations. Our finding that ILC3 express MHC II more highly towards the end of pregnancy could also imply that ILC3 may have roles other than IL-22 production at this time. Interactions of ILC3 with other immune cells in the uterus, akin to mucosal tissues elsewhere, also remain to be elucidated. These include their ability to present antigens to T cells [17, 22, 42, 43] and regulate myeloid cell functions [44] through effector molecules released into the local tissue microenvironment, mechanisms that will amplify the impact of this small immune cell population.

In summary, our data suggest that the primary role of uterine ILC3 may be in endometrial repair and regeneration, leading us to speculate that they may play a role in the pathogenesis of endometriosis. Furthermore, our comprehensive description of the dynamics and localization of uterine ILC3 across the healthy menstrual cycle and pregnancy provides a reference from which their role in gynaecological and obstetric pathologies can now be explored.

## Supplementary Material

kyaf004_suppl_Supplementary_Materials

## Data Availability

The data underlying this publication has been deposited at https://osf.io/c439u/

## References

[1] Vivier E , Artis D, Colonna M, Diefenbach A, Di Santo JP, Eberl G, et al Innate lymphoid cells: 10 years on. Cell 2018, 174, 1054–66. doi: https://doi.org/10.1016/j.cell.2018.07.017 30142344

[2] Male V , Moffett A. Natural killer cells in the human uterine mucosa. Annu Rev Immunol 2023, 41, 127–51. doi: https://doi.org/10.1146/annurev-immunol-102119-075119 36630598

[3] Doisne J-M , Balmas E, Boulenouar S, Gaynor LM, Kieckbusch J, Gardner L, et al Composition, development, and function of uterine innate lymphoid cells. J Immunol 2015, 195, 3937–45. doi: https://doi.org/10.4049/jimmunol.1500689 26371244 PMC4592103

[4] Vento-Tormo R , Efremova M, Botting RA, Turco MY, Vento-Tormo M, Meyer KB, et al Single-cell reconstruction of the early maternal–fetal interface in humans. Nature 2018, 563, 347–53. doi: https://doi.org/10.1038/s41586-018-0698-6 30429548 PMC7612850

[5] Vacca P , Montaldo E, Croxatto D, Loiacono F, Canegallo F, Venturini PL, et al Identification of diverse innate lymphoid cells in human decidua. Mucosal Immunol 2015, 8, 254–64. doi: https://doi.org/10.1038/mi.2014.63 25052762

[6] Montaldo E , Vacca P, Chiossone L, Croxatto D, Loiacono F, Martini S, et al Unique Eomes+ NK cell subsets are present in uterus and decidua during early pregnancy. Front Immunol 2016, 6, 646. doi: https://doi.org/10.3389/fimmu.2015.00646 27004067 PMC4794975

[7] Huhn O , Ivarsson MA, Gardner L, Hollinshead M, Stinchcombe JC, Chen P, et al Distinctive phenotypes and functions of innate lymphoid cells in human decidua during early pregnancy. Nat Commun 2020, 11, 381. doi: https://doi.org/10.1038/s41467-019-14123-z 31959757 PMC6971012

[8] Vazquez J , Chasman DA, Lopez GE, Tyler CT, Ong IM, Stanic AK. Transcriptional and functional programming of decidual innate lymphoid cells. Front Immunol 2020, 10, 3065. doi: https://doi.org/10.3389/fimmu.2019.03065 32038619 PMC6992589

[9] Trabanelli S , Gomez-Cadena A, Jandus C. Immunophenotyping of human innate lymphoid cells. In: McCoy Jr, JP (ed), Immunophenotyping, vol. 2032. New York, NY: Springer New York, 2019, 179–92. 10.1007/978-1-4939-9650-6_10 31522419

[10] Koprivica I , Stanisavljević S, Mićanović D, Jevtić B, Stojanović I, Miljković D. ILC3: a case of conflicted identity. Front Immunol 2023, 14, 1271699. doi: https://doi.org/10.3389/fimmu.2023.1271699 37915588 PMC10616800

[11] Male V , Hughes T, McClory S, Colucci F, Caligiuri MA, Moffett A. Immature NK cells, capable of producing IL-22, are present in human uterine mucosa. J Immunol 2010, 185, 3913–8. doi: https://doi.org/10.4049/jimmunol.1001637 20802153 PMC3795409

[12] Satoh-Takayama N. Heterogeneity and diversity of group 3 innate lymphoid cells: new cells on the block. Int Immunol 2016, 28, 29–34. doi: https://doi.org/10.1093/intimm/dxv054 26462712

[13] Dambaeva S , Schneiderman S, Jaiswal MK, Agrawal V, Katara GK, Gilman-Sachs A, et al Interleukin 22 prevents lipopolysaccharide-induced preterm labor in mice†. Biol Reprod 2018, 98, 299–308. doi: https://doi.org/10.1093/biolre/iox182 29315356 PMC6669419

[14] Gershater M , Romero R, Arenas-Hernandez M, Galaz J, Motomura K, Tao L, et al IL-22 plays a dual role in the amniotic cavity: tissue injury and host defense against microbes in preterm labor. J Immunol 2022, 208, 1595–615. doi: https://doi.org/10.4049/jimmunol.2100439 35304419 PMC8976826

[15] Croxatto D , Micheletti A, Montaldo E, Orecchia P, Loiacono F, Canegallo F, et al Group 3 innate lymphoid cells regulate neutrophil migration and function in human decidua. Mucosal Immunol 2016, 9, 1372–83. doi: https://doi.org/10.1038/mi.2016.10 26906405

[16] Hanna J , Goldman-Wohl D, Hamani Y, Avraham I, Greenfield C, Natanson-Yaron S, et al Decidual NK cells regulate key developmental processes at the human fetal-maternal interface. Nat Med 2006, 12, 1065–74. doi: https://doi.org/10.1038/nm1452 16892062

[17] Hepworth MR , Monticelli LA, Fung TC, Ziegler CGK, Grunberg S, Sinha R, et al Innate lymphoid cells regulate CD4+ T-cell responses to intestinal commensal bacteria. Nature 2013, 498, 113–7. doi: https://doi.org/10.1038/nature12240 23698371 PMC3699860

[18] Chen L , Youssef Y, Robinson C, Ernst GF, Carson MY, Young KA, et al CD56 expression marks human group 2 innate lymphoid cell divergence from a shared NK cell and group 3 innate lymphoid cell developmental pathway. Immunity 2018, 49, 464–76.e4. doi: https://doi.org/10.1016/j.immuni.2018.08.010 30193847 PMC6148384

[19] Lim AI , Li Y, Lopez-Lastra S, Stadhouders R, Paul F, Casrouge A, et al Systemic human ILC precursors provide a substrate for tissue ILC differentiation. Cell 2017, 168, 1086–100.e10. doi: https://doi.org/10.1016/j.cell.2017.02.021 28283063

[20] Whettlock EM , Woon EV, Cuff AO, Browne B, Johnson MR, Male V. Dynamic changes in uterine NK cell subset frequency and function over the menstrual cycle and pregnancy. Front Immunol 2022, 13, 880438. doi: https://doi.org/10.3389/fimmu.2022.880438 35784314 PMC9245422

[21] Arici A , Seli E, Senturk LM, Gutierrez LS, Oral E, Taylor HS. Interleukin-8 in the human endometrium. J Clin Endocrinol Metab 1998, 83, 1783–7. doi: https://doi.org/10.1210/jcem.83.5.4754 9589693

[22] Rao A , Strauss O, Kokkinou E, Bruchard M, Tripathi KP, Schlums H, et al Cytokines regulate the antigen-presenting characteristics of human circulating and tissue-resident intestinal ILCs. Nat Commun 2020, 11, 2049. doi: https://doi.org/10.1038/s41467-020-15695-x 32341343 PMC7184749

[23] Dutton EE , Withers DR. Identification of murine and human innate lymphoid cells in frozen tissue sections using immunofluorescence. In: Amarnath S (ed), Innate Lymphoid Cells, vol. 2121. New York, NY: Springer US, 2020, 51–8. 10.1007/978-1-0716-0338-3_5 32147785

[24] Pascual-Reguant A , Köhler R, Mothes R, Bauherr S, Hernández DC, Uecker R, et al Multiplexed histology analyses for the phenotypic and spatial characterization of human innate lymphoid cells. Nat Commun 2021, 12, 1737. doi: https://doi.org/10.1038/s41467-021-21994-8 33741932 PMC7979823

[25] Abramiuk M , Grywalska E, Małkowska P, Sierawska O, Hrynkiewicz R, Niedźwiedzka-Rystwej P. The role of the immune system in the development of endometriosis. Cells 2022, 11, 2028. doi: https://doi.org/10.3390/cells11132028 35805112 PMC9265783

[26] Izumi G , Koga K, Takamura M, Makabe T, Satake E, Takeuchi A, et al Involvement of immune cells in the pathogenesis of endometriosis. J Obstet Gynaecol Res 2018, 44, 191–8. doi: https://doi.org/10.1111/jog.13559 29316073

[27] Sugahara T , Tanaka Y, Hamaguchi M, Fujii M, Shimura K, Ogawa K, et al Reduced innate lymphoid cells in the endometrium of women with endometriosis. Am J Reprod Immunol 2022, 87, e13502. doi: https://doi.org/10.1111/aji.13502 34592011

[28] Freud AG , Keller KA, Scoville SD, Mundy-Bosse BL, Cheng S, Youssef Y, et al NKp80 defines a critical step during human natural killer cell development. Cell Rep 2016, 16, 379–91. doi: https://doi.org/10.1016/j.celrep.2016.05.095 27373165 PMC4970225

[29] Liu S , Sirohi K, Verma M, McKay J, Michalec L, Sripada A, et al Optimal identification of human conventional and nonconventional (CRTH2–IL7Rα–) ILC2s using additional surface markers. J Allergy Clin Immunol 2020, 146, 390–405. doi: https://doi.org/10.1016/j.jaci.2020.01.038 32032632 PMC7398840

[30] Bernink JH , Peters CP, Munneke M, te Velde AA, Meijer SL, Weijer K, et al Human type 1 innate lymphoid cells accumulate in inflamed mucosal tissues. Nat Immunol 2013, 14, 221–9. doi: https://doi.org/10.1038/ni.2534 23334791

[31] Boulenouar S , Doisne J-M, Sferruzzi-Perri A, Gaynor LM, Kieckbusch J, Balmas E, et al The residual innate lymphoid cells in NFIL3-deficient mice support suboptimal maternal adaptations to pregnancy. Front Immunol 2016, 7, 43. doi: https://doi.org/10.3389/fimmu.2016.00043 26925058 PMC4759249

[32] Marečková M , Garcia-Alonso L, Moullet M, Lorenzi V, Petryszak R, Sancho-Serra C, et al An integrated single-cell reference atlas of the human endometrium. Nat Genet 2024, 56, 1925–37. doi: https://doi.org/10.1038/s41588-024-01873-w 39198675 PMC11387200

[33] Guo Y , Chen Y, Liu L-B, Chang K-K, Li H, Li M-Q, et al IL-22 in the endometriotic milieu promotes the proliferation of endometrial stromal cells via stimulating the secretion of CCL2 and IL-8. Int J Clin Exp Pathol 2013, 6, 2011–20. 24133578 PMC3796222

[34] Kirkwood PM , Gibson DA, Shaw I, Dobie R, Kelepouri O, Henderson NC, et al Single-cell RNA sequencing and lineage tracing confirm mesenchyme to epithelial transformation (MET) contributes to repair of the endometrium at menstruation. eLife 2022, 11, e77663. doi: https://doi.org/10.7554/eLife.77663 36524724 PMC9873258

[35] Mulayim N , Palter SF, Kayisli UA, Senturk L, Arici A. Chemokine receptor expression in human endometrium1. Biol Reprod 2003, 68, 1491–5. doi: https://doi.org/10.1095/biolreprod.102.009639 12606476

[36] Eberl G , Sawa S. Opening the crypt: current facts and hypotheses on the function of cryptopatches. Trends Immunol 2010, 31, 50–5. doi: https://doi.org/10.1016/j.it.2009.11.004 20015688

[37] Dudakov JA , Hanash AM, Van Den Brink MRM. Interleukin-22: immunobiology and Pathology. Annu Rev Immunol 2015, 33, 747–85. 25706098 10.1146/annurev-immunol-032414-112123 PMC4407497

[38] Protopsaltis NJ , Liang W, Nudleman E, Ferrara N. Interleukin-22 promotes tumor angiogenesis. Angiogenesis 2019, 22, 311–23. doi: https://doi.org/10.1007/s10456-018-9658-x 30539314

[39] Ballard K , Seaman H, De Vries C, Wright J. Can symptomatology help in the diagnosis of endometriosis? Findings from a national case–control study—part 1. BJOG Int J Obstet Gynaecol 2008, 115, 1382–91. 10.1111/j.1471-0528.2008.01878.x 18715240

[40] McKinnon B , Mueller M, Montgomery G. Progesterone resistance in endometriosis: an acquired property? Trends Endocrinol Metab 2018, 29, 535–48. doi: https://doi.org/10.1016/j.tem.2018.05.006 29934050

[41] Biswas Shivhare S , Bulmer JN, Innes BA, Hapangama DK, Lash GE. Menstrual cycle distribution of uterine natural killer cells is altered in heavy menstrual bleeding. J Reprod Immunol 2015, 112, 88–94. doi: https://doi.org/10.1016/j.jri.2015.09.001 26398782

[42] Lyu M , Suzuki H, Kang L, Gaspal F, Zhou W, Goc J, et al; JRI Live Cell Bank. ILC3s select microbiota-specific regulatory T cells to establish tolerance in the gut. Nature 2022, 610, 744–51. doi: https://doi.org/10.1038/s41586-022-05141-x 36071169 PMC9613541

[43] Kedmi R , Najar TA, Mesa KR, Grayson A, Kroehling L, Hao Y, et al A RORγt+ cell instructs gut microbiota-specific Treg cell differentiation. Nature 2022, 610, 737–43. doi: https://doi.org/10.1038/s41586-022-05089-y 36071167 PMC9908423

[44] Pearson C , Thornton EE, McKenzie B, Schaupp A-L, Huskens N, Griseri T, et al ILC3 GM-CSF production and mobilisation orchestrate acute intestinal inflammation. eLife 2016, 5, e10066. doi: https://doi.org/10.7554/eLife.10066 26780670 PMC4733039

[45] Stricker R , Eberhart R, Chevailler M-C, Quinn FA, Bischof P, Stricker R. Establishment of detailed reference values for luteinizing hormone, follicle stimulating hormone, estradiol, and progesterone during different phases of the menstrual cycle on the Abbott ARCHITECT® analyzer. Clin Chem Lab Med 2006, 44, 883–7. doi: https://doi.org/10.1515/CCLM.2006.160 16776638

[46] Cocker ATH , Whettlock EM, Browne B, Lai PF, Li JKH, Sivarajasingam SP, et al Isolation of single cells from human uterus in the third trimester of pregnancy: myometrium, decidua, amnion and chorion. Oxf Open Immunol 2022, 3, iqac010. doi: https://doi.org/10.1093/oxfimm/iqac010 36846559 PMC9914580

[47] Papuchova H , Kshirsagar S, Xu L, Bougleux Gomes HA, Li Q, Iyer V, et al Three types of HLA-G+ extravillous trophoblasts that have distinct immune regulatory properties. Proc Natl Acad Sci U S A 2020, 117, 15772–7. doi: https://doi.org/10.1073/pnas.2000484117 32581122 PMC7355041

[48] Brummelman J , Haftmann C, Núñez NG, Alvisi G, Mazza EMC, Becher B, et al Development, application and computational analysis of high-dimensional fluorescent antibody panels for single-cell flow cytometry. Nat Protoc 2019, 14, 1946–69. doi: https://doi.org/10.1038/s41596-019-0166-2 31160786

[49] Liechti T , Weber LM, Ashhurst TM, Stanley N, Prlic M, Van Gassen S, et al An updated guide for the perplexed: cytometry in the high-dimensional era. Nat Immunol 2021, 22, 1190–7. doi: https://doi.org/10.1038/s41590-021-01006-z 34489590

