# Supplementary material for: Dynamic roles of ILC3 in endometrial repair and regeneration
Source: Discov Immunol. 2025 Mar 26;4(1):kyaf004. doi: 10.1093/discim/kyaf004 (PMC12038238; doi:10.1093/discim/kyaf004)
Supplement: kyaf004_suppl_Supplementary_Materials [file kyaf004_suppl_supplementary_materials.pdf]

Supplementary Figures

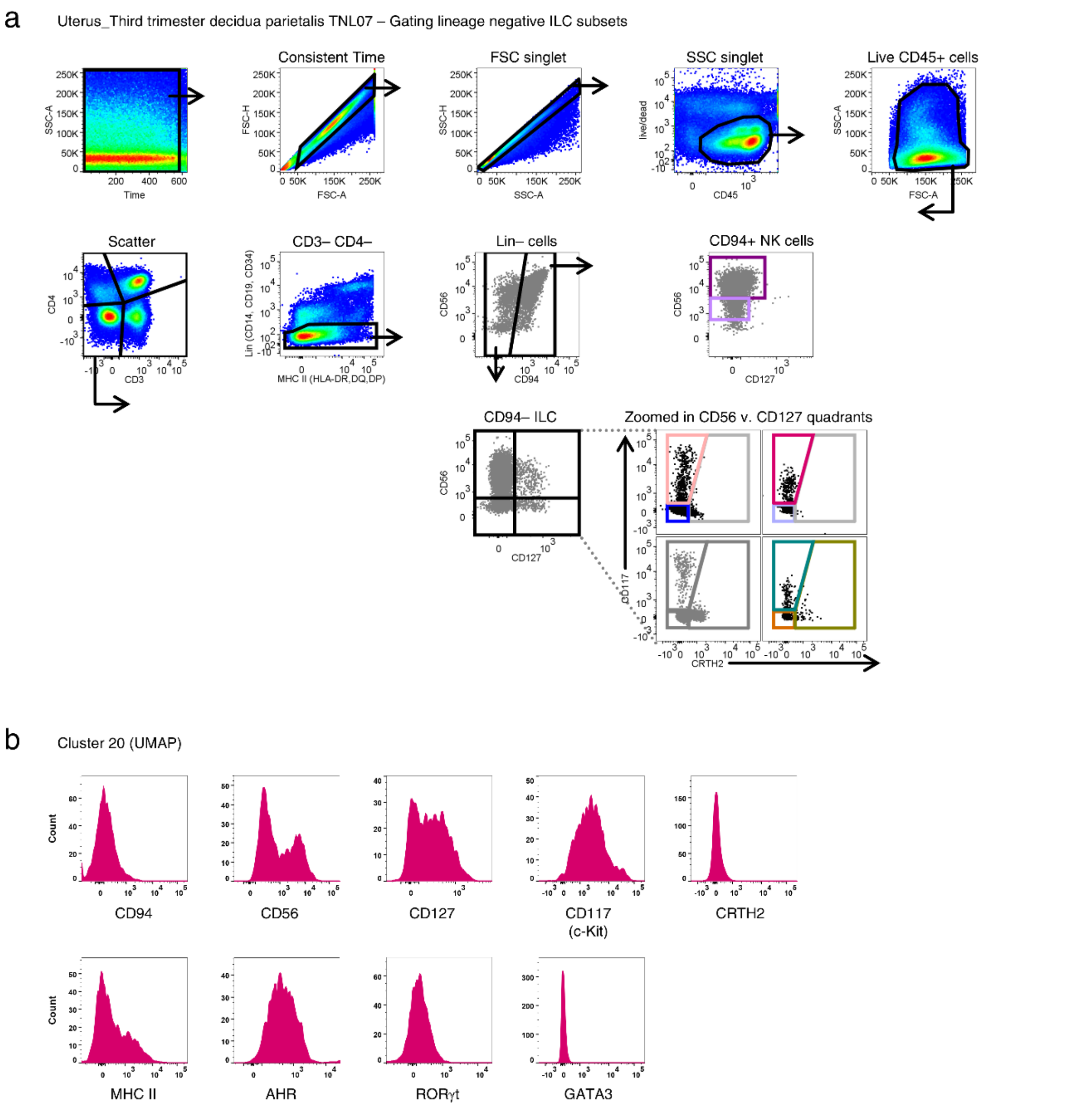

Supplementary Figure 1. **A:** Complete Flow cytometric gating strategy to identify ILC subsets among CD45+ Lin– immune cells isolated from uterine mucosal tissue and matched peripheral blood at different stages of the human reproductive life cycle. **B:** Marker expression by cluster 20 cells from the UMAP landscape.

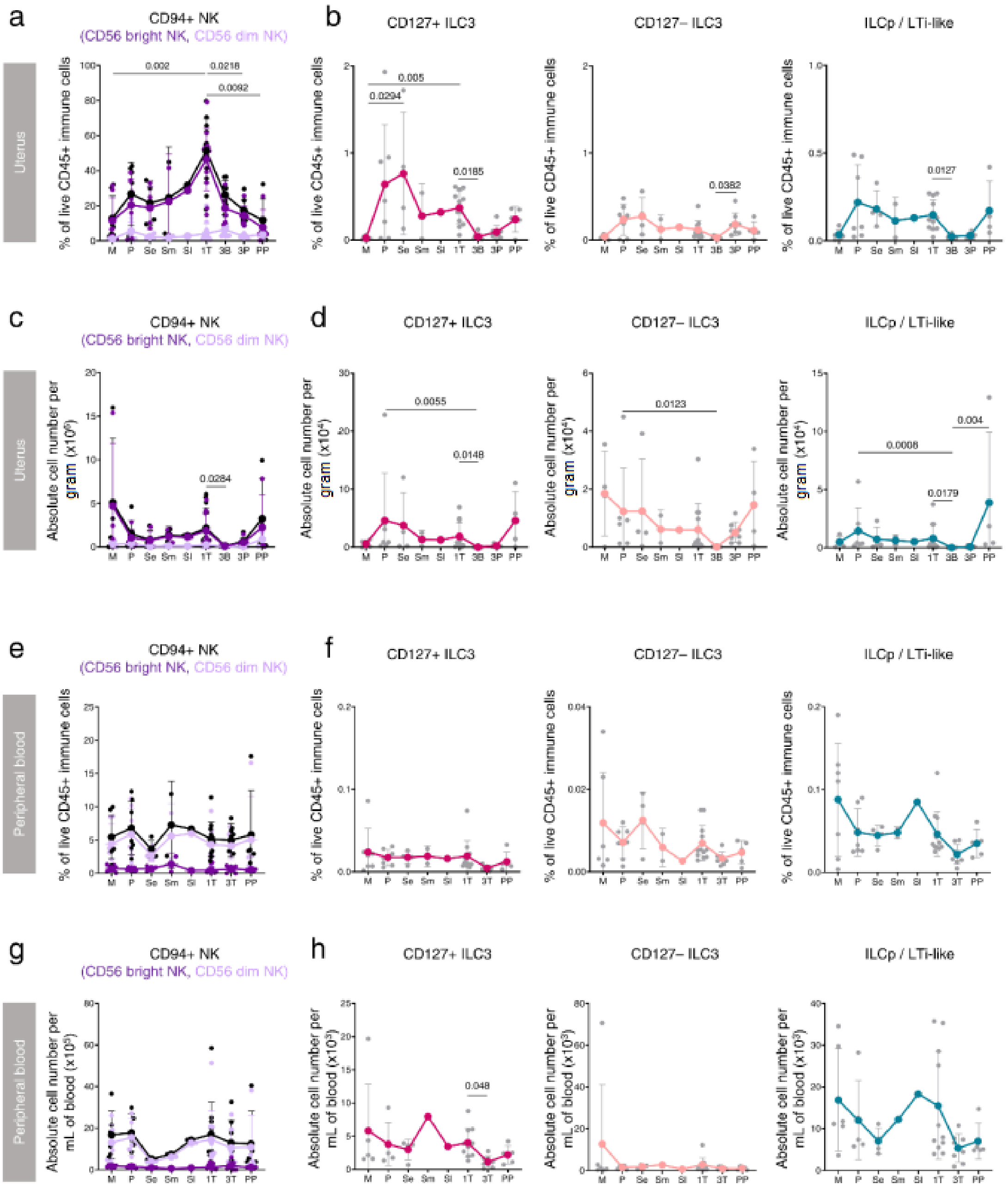

Supplementary Figure 2. Uterine and matched peripheral blood ILC3 may vary during the secretory menstrual phase

A: Frequency of NK cells in the uterus across the menstrual cycle and pregnancy. **B:** Frequency of ILC3 subsets in the uterus across the menstrual cycle and pregnancy. **C:** Absolute number of NK cells per gram of uterine tissue. **D:** Absolute number of ILC3 subsets per gram of uterine tissue. **E:** Frequency of NK cells in blood across the menstrual cycle and

pregnancy. **F:** Frequency of ILC3 subsets in blood across the menstrual cycle and pregnancy. **G:** Absolute number of NK cells per mL of blood. **H:** Absolute number of ILC3 subsets per mL of blood. A Kruskal-Wallis statistical test for significance was performed with a Dunn's correction for multiple hypotheses testing. Menses (M, n = 7), proliferative (P, n = 7), secretory (Se- early, n = 4; Sm – middle, n = 2 uterus, n = 1 blood; Sl – late, n = 1), first trimester (1T, n = 12), third trimester (3T blood, n = 8), decidua basalis (3B, n = 8) or parietalis (3P, n = 8), and postpartum (PP, n = 5).

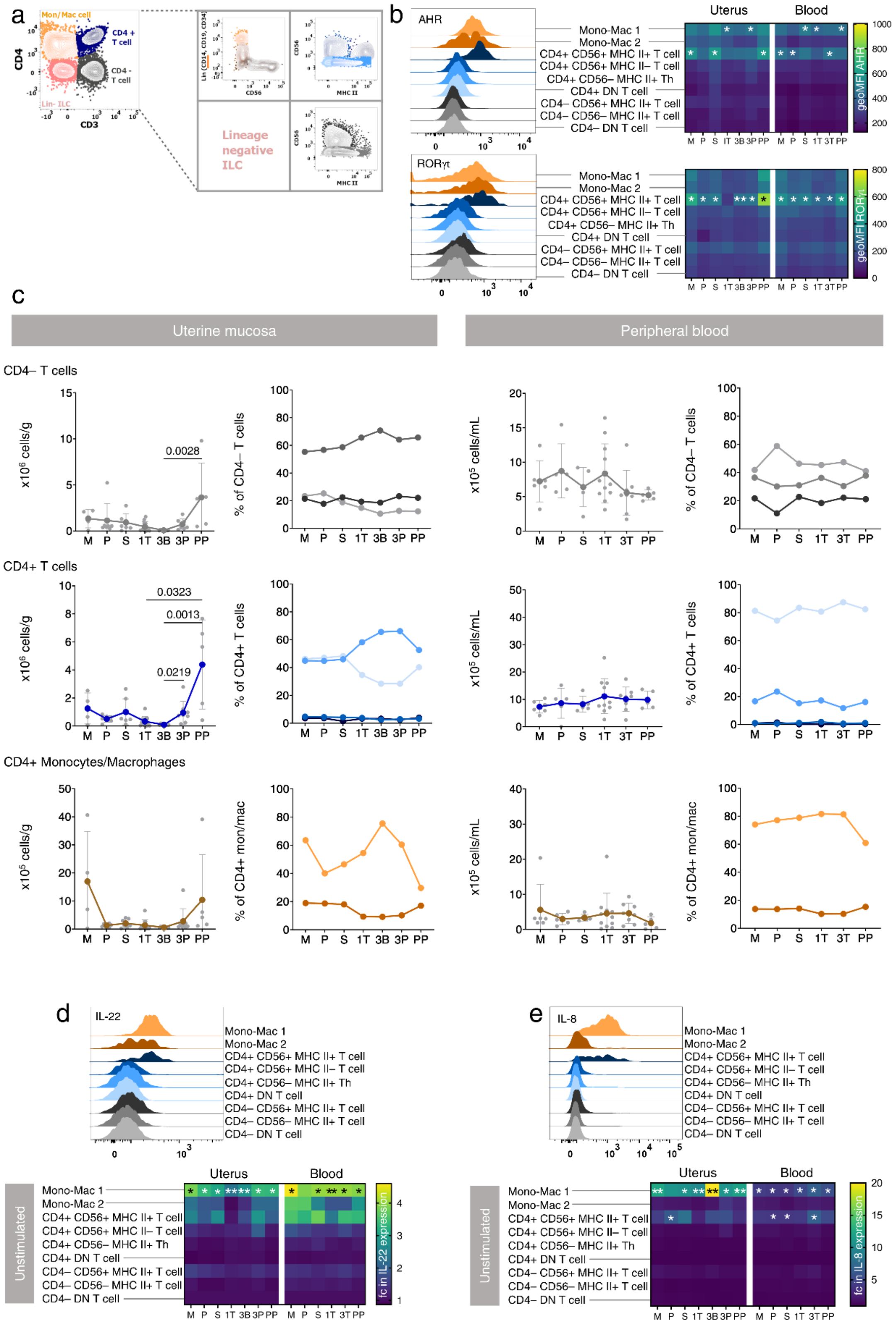

Supplementary Figure 3. **A:** Flow cytometric gating strategy to identify non-ILC immune cells among CD45+ immune cells isolated from uterine mucosal tissue and matched peripheral

blood at different stages of the human reproductive life cycle. **B:** Expression of transcription factors AHR and RORγt by non-ILC immune cells in both the uterus and blood. **C:** Representative frequencies and absolute number of non-ILC immune cell subpopulations in the uterus and blood at different of the human reproductive life cycle. **D:** Intracellular IL-22 production by non-ILC immune cells. **E:** Intracellular IL-8 production by non-ILC immune cells. Significance was determined via Z score transformation (one-tailed), * $p < 0.05$, ** $p < 0.01$. Stages examined included the menses (M, n = 5), proliferative (P, n = 6 uterus, n = 5 blood), secretory (S, n = 7), first trimester (1T, n = 11 uterus, n = 10 blood), third trimester (3T blood, n = 5), decidua basalis (3B, n = 5) or parietalis (3P, n = 5), and postpartum (PP, n = 3) stages. DN: CD56– MHC II–; Mono-Mac1/2: Monocytes in blood or Macrophages in the uterus; Th – Helper T lymphocytes.

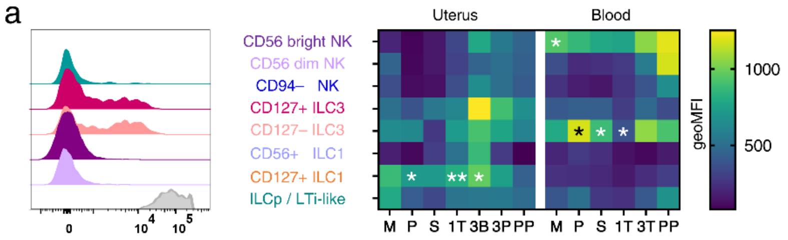

Supplementary Figure 4. **A:** Histogram and heatmap representation of MHC II expression by uterine and peripheral blood ILC3. Z score transformed statistics (one-tailed), * p < 0.05, ** p < 0.01. Stages examined included the menses (M, n = 5), proliferative (P, n = 6 uterus, n = 5 blood), secretory (S, n = 7), first trimester (1T, n = 11 uterus, n = 10 blood), third trimester (3T blood, n = 5), decidua basalis (3B, n = 5) or parietalis (3P, n = 5), and postpartum (PP, n = 3) stages. Internal MHC II positive control (grey) is represented by Mono-Mac 1 cells (see gating in Supplementary Figure 4).

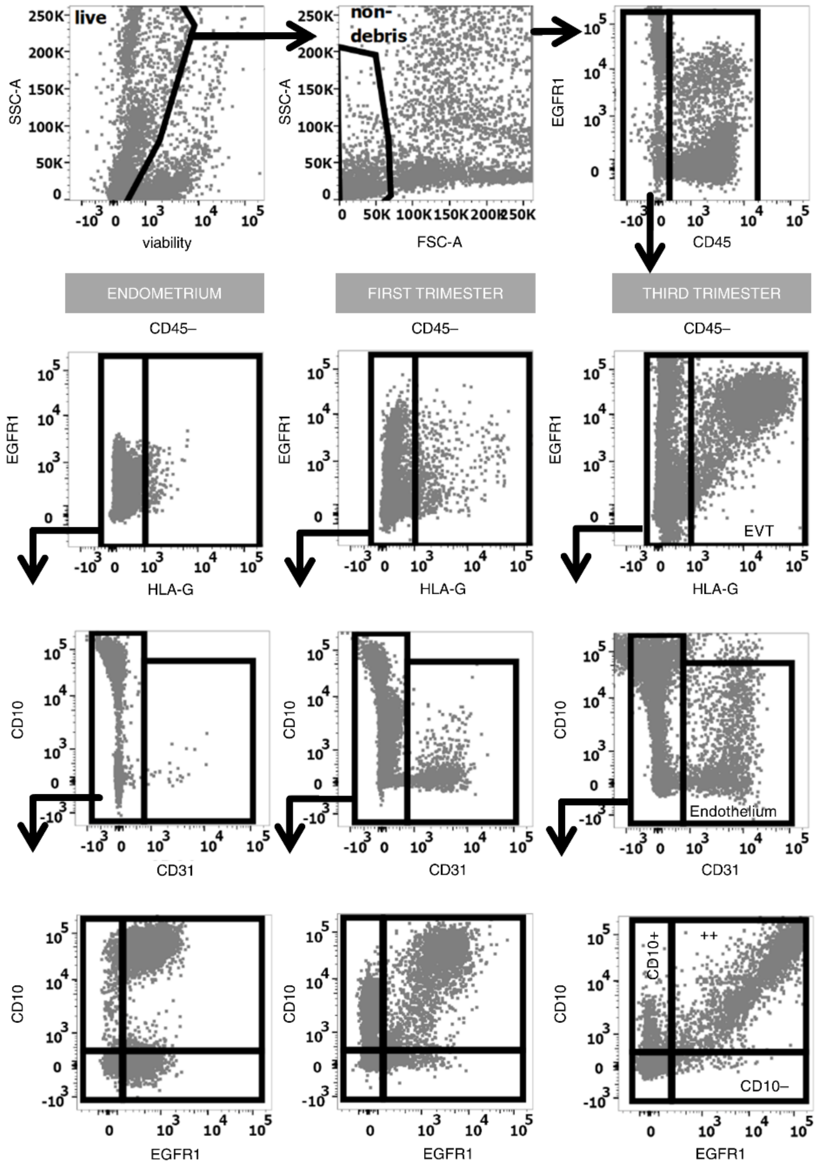

Supplementary Figure 5. Complete flow cytometric gating strategy for non-immune cells from the lining of the uterus at different stages of the human reproductive life cycle. Non-immune cells were identified by gating live CD45 negative cells excluded of debris (low scatter). Among CD45 negative cells, HLA-G positive expression distinguished pregnancy-related epithelial cells (trophoblasts) from all other cells, which lacked HLA-G expression. CD31 marked endothelial cells among HLA-G negative cells. Further subsets of epithelial and stromal cells were identified by their expression of EGFR1 and CD10; CD10+ stroma, EGFR1+ epithelium, and CD10+ EGFR1+ epithelium (++).

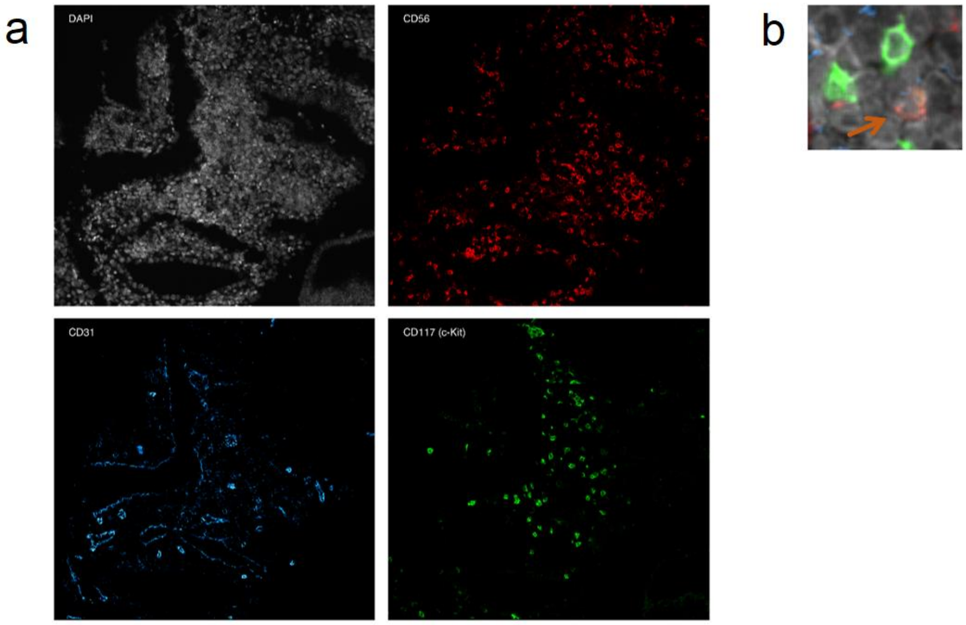

Supplementary Figure 6. **A:** Representative single-colour frames of immunofluorescence. **B:** Magnification of a representative ILC3, showing coexpression of CD56 (green) and CD117 (red).

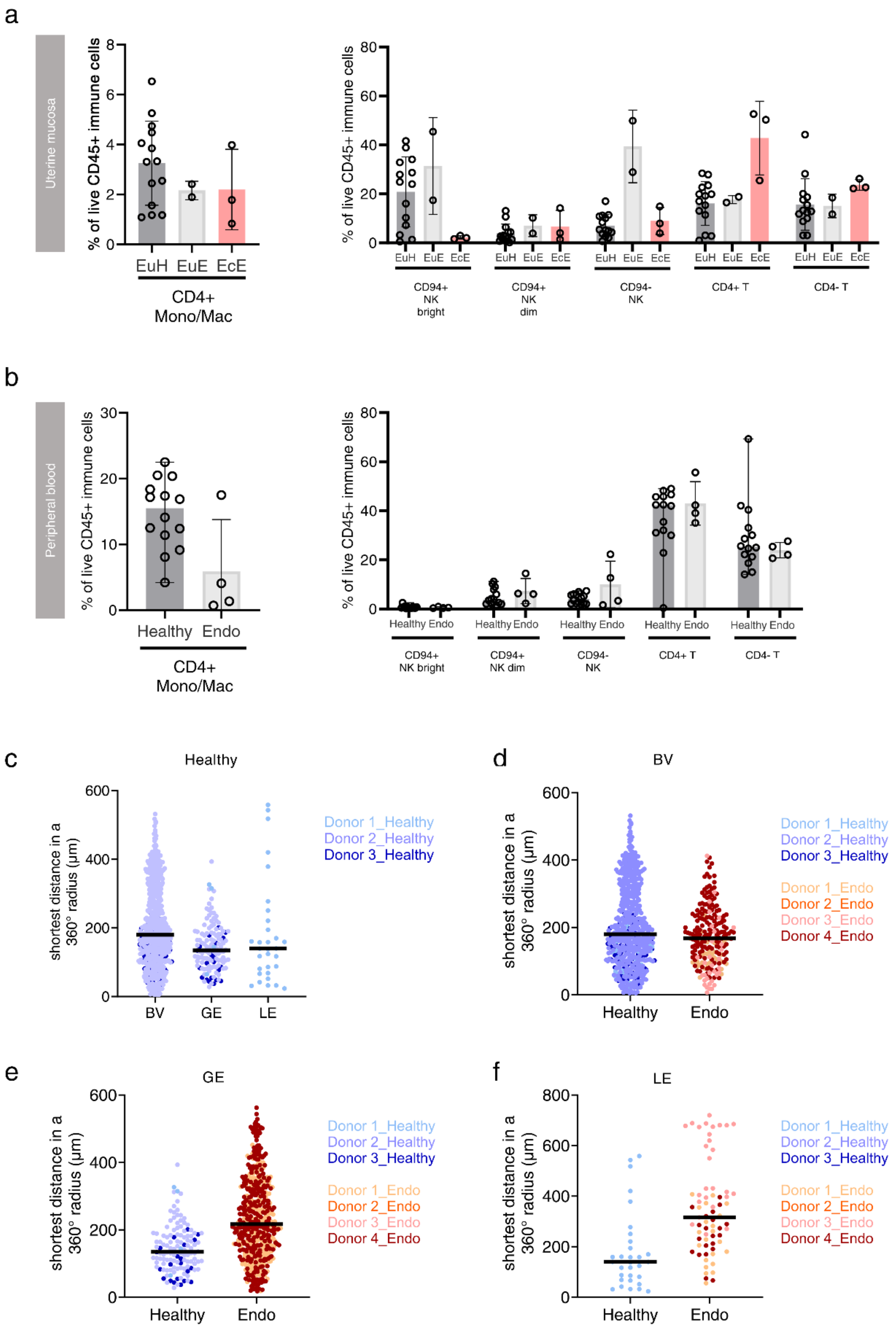

Supplementary Figure 7. **A:** Frequency of CD4+ monocytes/macrophages, NK cells, and T cells among total live CD45+ immune cells isolated from eutopic endometrium in healthy

donors (EuH, proliferative (P, n = 7), secretory (S, n = 7), eutopic endometrium in donors with endometriosis (EuE, proliferative (P, n = 1), secretory (S, n = 1) and ectopic endometrium in donors with endometriosis (EcE, proliferative (P, n = 1), secretory (S, n = 1), undefined (n = 1). **B:** Frequency of CD4+ monocytes/macrophages, NK cells, and T cells in the blood of healthy donors and donors with endometriosis (Healthy donors, proliferative (P, n = 7), secretory (S, n = 7; Endometriosis donors, proliferative (P, n = 1), secretory (S, n = 2), undefined (n = 1). **C:** The distance between each NK cell and their surrounding endometrial structural component were determined using Fiji (median shown). Differences between each NK cell and their nearest (D) blood vessel, (E) glandular epithelial cell or (F) luminal epithelium in healthy donors (data duplicated from Supplementary Figure 7c) and endometriosis patients are indicated (nested t test, two-tailed).

Supplementary Tables

Supplementary Table 1. Demographic information for eutopic endometrial samples collected from healthy non-pregnant donors at different phases of the menstrual cycle.

Sample ID	Age	Parity	Secretory sub-phase	Cycle day	Cycle length	Serum progesterone (nmol/L)	Phase assigned	Assessment	Comorbidity / Medication	Self-assigned ethnicity
E18	27	nulliparous		10	30	1	Proliferative	Phenotype	None	Caucasian
E20	24	nulliparous	mid	24	25	47	Secretory	Phenotype	None	Caucasian
E22	29	nulliparous		7	28	<1	Proliferative	Phenotype	None	Caucasian
E24	32	multiparous		n/a	n/a	<1	Postpartum	Phenotype	None	Indian
E25	29	nulliparous	early	20	35	7	Secretory	Phenotype	None	Caucasian
E26	36	multiparous		1	28	3	Menses	Phenotype	None	Caucasian
E27	25	nulliparous	early	13	21	5	Secretory	Phenotype	None	Caucasian
E28	23	nulliparous		2	28	1	Menses	Phenotype	None	Caucasian
E36	23	nulliparous		1	29	1	Menses	Phenotype	None	Afro-Caribbean
E37	40	multiparous	mid	21	28	28	Secretory	Phenotype	None	Caucasian

E38	24	nulliparous	early	17	34	6	Secretory	Phenotype	None	Caucasian
E41	25	nulliparous	early	17	28	NT	Secretory	Function	4 months COCP withdrawn	Caucasian
E43	33	multiparous	mid	19	28	22	Secretory	Function	none	Caucasian
E44	38	multiparous		1	32	1	Menses	Phenotype / Function	multiple miscarriages (x7)	Caucasian
E45	24	nulliparous	early	19	28	8	Secretory	Phenotype	sertraline, lamotrigine, pregabalin	Caucasian
E47	31	multiparous		10	28	5	Proliferativ e	Phenotype	None	Afro-Caribbean
E48	23	nulliparous		8	28	<1	Proliferativ e	Phenotype	6 months COCP withdrawn	Caucasian
E49	32	multiparous	late	28	31	8	Secretory	Function	Breastfeeding	Caucasian
E50	19	nulliparous	late	27	28	20	Secretory	Function	None	Caucasian
E52	32	nulliparous	early	17	28	13	Secretory	Function	None	Afro-Caribbean
E53	40	nulliparous		11	28	1	Proliferativ e	Phenotype	Recent UTI: (completed Trimethoprim), covid-vaccinated 2 weeks prior	Caucasian
E56	36	multiparous	late	26	30	32	Secretory	Phenotype	None	Pakistan
E57	37	multiparous		n/a	n/a	3	Postpartum	Phenotype	None	North African
E59	29	multiparous		n/a	n/a	FT	Postpartum	Phenotype	None	Pakistan
E61	22	nulliparous	early	17	28	1	Secretory	Receptor	None, low serum progesterone	Caucasian
E62	33	nulliparous	late	27	30	34	Secretory	Receptor	None	Caucasian

E63	23	nulliparous		7	36	NT	Proliferative	Function / Receptor	None	Afro-Caribbean
E64	32	nulliparous	late	28	28	14	Secretory	Receptor	Spironolactone for Acne	Caucasian
E65	28	multiparous		n/a	n/a	<1	Postpartum	Function / Receptor	None	Asian - Other
E66	24	nulliparous		7	28	1	Proliferative	Function	None	Caucasian
E68	36	multiparous		n/a	n/a	NT	Postpartum	Phenotype / Function / Receptor	None	Afro-Caribbean
E70	34	multiparous		13	28	8	Proliferative	Phenotype / Function / Receptor	None	Caucasian
E73	34	nulliparous		8	28	<1	Proliferative	Phenotype / Function	High BMI, previous herpes	Caucasian
E74	24	nulliparous		1	28	1	Menses	Phenotype / Function	Anti-depressant	Asian - Other
E75	27	nulliparous		7	28	<1.6	Proliferative	Function	None	Asian
E76	26	nulliparous		4	28	<1.6	Menses	Phenotype / Function	None	Caucasian
E77	21	nulliparous	late	29	35	1.6	Secretory	Receptor	Chronic pain, long cycle (query PCOS), low serum progesterone	Caucasian
E79	23	nulliparous		1	28	NT	Menses	Function	None	Caucasian
E80	25	nulliparous		5	28	NT	Menses	Phenotype / Function	None	Caucasian
E83	24	nulliparous	late	31	31	NT	Secretory	Function	None	Caucasian

E84	30	nulliparous		10	28	NT	Proliferativ e	Function	None	Other
E85	33	multiparous		n/a	n/a	NT	Postpartum	Phenotype / Function	None	Caucasian

<u>Key</u>

Parity: – indicates if patient has had a previous livebirth (birth of a live offspring > 24 weeks of gestation). Nulliparous refers to patients who were not pregnant at point of samples collection and had no previous livebirths, and this may include previous miscarriages or termination of pregnancies. Primiparous refers to patients who were pregnant at point of sample collection but had no previous livebirths, and this may include previous miscarriages and termination of pregnancies. Multiparous refers to patients who had previous livebirths.

NT – not tested; FT – failed test (irrelevant for assigned samples where values are expected to be low or negative).

PCOS – polycystic ovarian syndrome

BMI – body mass index

COCP – combined oral contraceptive pill

UTI – urinary tract infection

Secretory phases: early (days 14-19), mid (days 19-24), late (days 24-28)

Supplementary Table 2. Demographic information for eutopic endometrial samples and ectopic lesions collected from donors with endometriosis.

sample ID	Age	Parity	Cycle day	Cycle length	Serum progesterone (nmol/L)	Phase assigned	Assessment	Tissue type	Comorbidity / Medication	Self-assigned ethnicity
E34	32	nulliparous	14	28	38	Secretory	Phenotype / Function	Eutopic endometrium	none	Caucasian
E58	34	nulliparous	6	28	<1	Proliferative	Phenotype	Eutopic endometrium	none	Caucasian
ACE01	27	nulliparous	22	30	NT	Secretory	Phenotype	Ectopic ovarian endometrioma (OE)	none	Middle eastern
ACE02	34	nulliparous	n/a	n/a	NT	Contraceptive altered	Phenotype	Ectopic peritoneal nodule (NO)	Patient on continuous combined oral contraceptive pill	Middle eastern
ACE04	31	nulliparous	7	30	NT	Proliferative	Phenotype / Function	Ectopic ovarian endometrioma (OE)	Infertility (having had five failed previous	Caucasian

									embryo transfers)	
ACE05	45	nulliparous	32	32	NT	Late Secretory	Phenotype	Ectopic ovarian endometrioma (OE)	none	Caucasian

Supplementary Table 3. Demographic information for eutopic endometrial samples from the EXPPECT biobank (University of Edinburgh). Samples were collected with informed consent from donors with endometriosis of known fertility status, and healthy control donors without endometriosis. Healthy control donors may have a benign gynecological condition, such as pain, also with confirmed fertility status. Samples were derived from women of reproductive age, fixed in 4% paraformaldehyde, dehydrated in 70% ethanol and embedded into paraffin wax blocks for storage.

sample ID	Tissue type	Endometriosis stage: revised American Society for Reproductive Medicine (rASRM) classification	Fertility status checked	Phase assigned	Assessment	Biobank year
1083	Eutopic endometrium_Control	na	fertile	Early Secretory	Immunofluorescence	2016
3266	Eutopic endometrium_Control	na	fertile	Early Secretory	Immunofluorescence	2016
4022	Eutopic endometrium_Control	na	fertile	Mid-Secretory	Immunofluorescence	2017
1076	Eutopic endometrium_Endometriosis	Stage 4 (>40)-Severe	fertile	Early Secretory	Immunofluorescence	2016
1033	Eutopic endometrium_Endometriosis	Stage 4 (>40)-Severe	fertile	Early Secretory	Immunofluorescence	2017

1805	Eutopic endometrium_Endometriosis	Stage 4 (>40)-Severe	fertile	Mid-Secretory	Immunofluorescence	2019
3267	Eutopic endometrium_Endometriosis	Stage 4 (>40)-Severe	infertile	Early Secretory	Immunofluorescence	2016
1658	Eutopic endometrium_Endometriosis	Stage 4 (>40)-Severe	infertile	Early Secretory	Immunofluorescence	2017
1262	Eutopic endometrium_Endometriosis	Stage 4 (>40)-Severe	infertile	Late Secretory	Immunofluorescence	2016

Supplementary Table 4. Demographic information for decidual samples collected from donors during different pregnancy stages.

**sample ID**	**Age**	**Parity**	**Gestation**	**Reproductive Stage**	**Delivery**	**Assessment**	**Pregnancy-related Comorbidity / Medication**	**Self-assigned ethnicity**
TOP10	24	Multiparous	8+6	1T	Personally-requested termination	Phenotype / Function	None	Caucasian
TOP12	20	Primiparous	6+6	1T	Personally-requested termination	Phenotype / Function / Receptor	None	Caucasian
TOP13	32	Multiparous	10+4	1T	Personally-requested termination	Phenotype / Function / Receptor	high BMI	Caucasian
TOP14	25	Primiparous	11+5	1T	Personally-requested termination	Phenotype / Function / Receptor	None	Asian_Indian
TOP15	22	Primiparous	8+3	1T	Personally-requested termination	Phenotype / Function / Receptor	Cannabis use, chronic ITP	Caucasian

TOP16	21	Multiparous	13/0	1T	Personally-requested termination	Phenotype / Function / Receptor	high BMI	Caucasian
TOP17	33	Multiparous	12/6	1T	Personally-requested termination	Phenotype / Function / Receptor	None	Caucasian
TOP19	30	Multiparous	6/0	1T	Personally-requested termination	Phenotype / Function	High BMI	Caucasian
TOP21	40	Multiparous	8/6	1T	Personally-requested termination	Phenotype / Function	On allopurinol (self-medicating)	Asian_Indian
TOP22	40	Multiparous	10+5	1T	Personally-requested termination	Phenotype / Function	SLE	Mixed
TOP23	26	Multiparous	6/0	1T	Personally-requested termination	Phenotype / Function	Cervical dyskaryosis (CIN 2)	Caucasian
TOP24	34	Multiparous	9+6	1T	Personally-requested termination	Phenotype / Function	asthma	Caucasian
TOP25	19	Primiparous	10+0	1T	Personally-requested termination	Phenotype / Function	Depression on sertraline and circadin	Caucasian
AC_TNL01	35	Multiparous	37+0	3T	Elective caesarean	Phenotype/Receptors	Pregnancy induced hypertension	White – Any other White background
AC_TNL02	30	Primiparous	38+0	3T	Elective caesarean	Phenotype	Ulcerative colitis (Mesalazine), bile acid malabsorption (cholestagel)	White – British
AC_TNL03	39	Multiparous	39+0	3T	Elective caesarean	Phenotype	none	White – Any other White background

AC_TNL04	35	Multiparous	39+0	3T	Elective caesarean	Function	none	Any Other Ethnic Group
AC_TNL05	35	Primiparous	39+0	3T	Elective caesarean	Phenotype	PCOS	White – British
AC_TNL06	40	Primiparous	38+6	3T	Elective caesarean	Phenotype	none	
AC_TNL07	41	Multiparous	39+3	3T	Elective caesarean	Phenotype	query hypothyroidism; loop exciscion of cervix to remove pre-cancerous cells	White other (Eastern European)
AC_TNL09	34	Primiparous	39+0	3T	Elective caesarean	Function / Receptors	hypothyroidism	Caucasian
AC_TNL10	41	Primiparous	39+1	3T	Elective caesarean	Receptors	Hashimoto's thyroiditis; loop excision of cervix to remove pre-cancerous cells	White British
AC_TNL11	36	Primiparous	39+0		Elective caesarean	Function / Receptors	previous shoulder operation	White British
AC_TNL13	31	Multiparous	39+0	3T	Elective caesarean	Function	Aortic coarctation	White British
AC_TNL14	41	Primiparous	39+0	3T	Elective caesarean	Phenotype / Function	Aspirin, cyclogest	White – Any other White background
AC_TNL15	41	Primiparous	39+3	3T	Elective caesarean	Phenotype / Function	none	Any Other Ethnic Group

Key – as above.

Supplementary Table 5. List of fluorescent-conjugated antibodies used in flow cytometry experiments

Antibody	Clone	Fluorophore	Dilution	Host animal	Manufacturer	Catalog #	RRID
**ILC3 backbone panel**							
CD3	SK7	APC-eFluor 780	1/200	Ms	eBioscience	47-0036	AB_10718679
CD4	OKT4	Brilliant Violet 785	1/100	Ms	Biolegend	317441	AB_2561365
CD14	63D3	FITC	1/100	Ms	Biolegend	367115	AB_2571928
CD19	HIB19	FITC	1/100	Ms	Biolegend	302205	AB_314235
CD34	561	FITC	1/100	Ms	Biolegend	343603	AB_1732030
CD45	2D1	Alexa Fluor 700	1/100	Ms	Biolegend	368513	AB_2566373
CD56	NCAM16.2	Brilliant Violet 510	1/200	Ms	BD Biosciences	563041	AB_2732786
CD94	DX22	PerCP-Cy5.5	1/200	Ms	Biolegend	305514	AB_2565522
CD117	104D2	PE-Dazzle 594	1/200	Ms	Biolegend	313225	AB_2566212
CD127	HIL-7R-M21	Alexa Fluor 647	1/20	Ms	BD Biosciences	558598	AB_647113
CD294 (CRTH2)	BM16	Brilliant Violet 605	1/50	Rat	Biolegend	350121	AB_2566759

HLA-DR,DQ,DP	Tu39	Brilliant Violet 711	1/100	Ms	BD Biosciences	740784	AB_2740447
**ILC phenotypic transcription factors**							
RORγt	Q21-559	Brilliant Violet 650	1/20	Ms	BD Biosciences	563424	AB_2738197
AHR	FF3399	PE	1/20	Ms	eBioscience	12-9854	AB_2572745
GATA3	L50-823	PE-Cy7	1/25	Ms	BD Biosciences	560405	AB_1645544
**ILC3 functional effector molecules**							
IL-8	G265-8	PE	1/100	Ms	BD Biosciences	554720	AB_395529
IL-22	22URTI	PE-Cy7	1/20	Ms	eBioscience	25-7229	AB_10853659
**Effector molecule receptor panel**							
CD10	HI10a	Brilliant Violet 785	1/100	Ms	Biolegend	312237	AB_2860829
CD31 (PECAM-1)	WM-59 (WM59)	APC-eFluor 780	1/100	Ms	eBioscience	47-0319	AB_10730582
CXCR1	5A12	Brilliant Violet 711	1/100	Ms	BD Biosciences	743423	AB_2741496
CXCR2	5E8/CXCR2	PE-Dazzle 594	1/100	Ms	Biolegend	320722	AB_2750215
EGFR1	AY13	PE-Cy7	1/200	Ms	Biolegend	352910	AB_2562159
HLA-G	MEM/9	FITC	1/25	Ms	Invitrogen	MA1-19591	AB_1076722
IL10Rβ	90220	Alexa Fluor 647	1/20	Ms	R&D Systems	FAB874R-100UG	na
IL22Rα1	305405	PE	1/20	Ms	R&D Systems	FAB2770P	AB_2124369

Supplementary Table 6. List of reagents for fluorescence microscopy

A: Host origin and dilution of primary antibodies used in fluorescence microscopy.

Antibody	Clone	Conjugation	Dilution	Origin	Manufacturer	Retrieval solution	Catalog #	RRID
Anti-human CD117 (c-Kit)	YR145	unconjugated	1/200	rabbit	Abcam	Tris-EDTA pH9.0	ab32363	AB_731513
Anti-human CD56	123C3	unconjugated	1/500	mouse	Invitrogen	EDTA pH8.0	18–0152	AB_138599
Anti-human CD31	C31.3 + JC/70A	unconjugated	1/200	mouse	Abcam	Citrate pH6.0	ab199012	AB_2756834; AB_307284

B: Host origin and dilution of secondary antibodies used in fluorescence microscopy.

Antibody	Clone	Conjugation	Dilution	Origin	Manufacturer	Catalog #	RRID
Anti-rabbit IgG	polyclonal	HRP	1/500	donkey	Jackson Immuno Research	711-036-152	AB_2340590
Anti-mouse IgG	polyclonal	HRP	1/500	donkey	Jackson Immuno Research	715-036-150	AB_2340773

C: Fluorescent dyes used for marker detection under catalysis of horse-radish peroxidase.

Antibody	Manufacturer	Catalog #
Opal 520 (green)	Akoya Biosciences	FP1487001KT
Opal 570 (red)	Akoya Biosciences	FP1488001KT
Opal 650 (blue)	Akoya Biosciences	FP1496001KT
1X Diluent	Akoya Biosciences	FP1498
